# The ICF2 gene *Zbtb24* specifically regulates the differentiation of B1 cells via promoting heme synthesis

**DOI:** 10.1186/s11658-024-00641-2

**Published:** 2024-09-14

**Authors:** He Gao, Ying Zhao, Sai Zhao, Xiao-Qiu Dai, Xiao-Yuan Qin, Wei-Long Zheng, Ting-Ting He, Nan Zhang, Can Zhu, Hong-Min Wang, Wen Pan, Xue-Mei Zhu, Xiao-Ming Gao, Jian-Feng Dai, Fang-Yuan Gong, Jun Wang

**Affiliations:** 1 Institutes of Biology and Medical Sciences, MOE Key Laboratory of Geriatric Diseases and Immunology, Jiangsu Key Laboratory of Infection and Immunity, Suzhou Medical College of Soochow University, Suzhou, 215123 China; 2 Department of Pathophysiology, School of Basic Medical Sciences, Suzhou Medical College of Soochow University, Suzhou, 215123 China; 3 Department of Immunology, School of Basic Medical Sciences, Suzhou Medical College of Soochow University, Suzhou, 215123 China

**Keywords:** ICF2, *Zbtb24*, B1 cells, Plasma cell differentiation, Heme synthesis

## Abstract

**Background:**

Loss-of-function mutations of *ZBTB24* cause immunodeficiency, centromeric instability, and facial anomalies syndrome 2 (ICF2). ICF2 is a rare autosomal recessive disorder with immunological defects in serum antibodies and circulating memory B cells, resulting in recurrent and sometimes fatal respiratory and gastrointestinal infections. The genotype–phenotype correlation in patients with ICF2 indicates an essential role of ZBTB24 in the terminal differentiation of B cells.

**Methods:**

We used the clustered regularly interspaced short palindromic repeats (CRISPER)/Cas9 technology to generate B cell specific *Zbtb24*-deficient mice and verified the deletion specificity and efficiency by quantitative polymerase chain reaction (Q-PCR) and western blotting analyses in fluorescence-activated cell sorting (FACS)-sorted cells. The development, phenotype of B cells and in vivo responses to T cell dependent or independent antigens post immunization were analyzed by flow cytometry and enzyme-linked immunosorbent assay (ELISA). Adoptive transfer experiment in combination with in vitro cultures of FACS-purified B cells and RNA-Seq analysis were utilized to specifically determine the impact of *Zbtb24* on B cell biology as well as the underlying mechanisms.

**Results:**

*Zbtb24* is dispensable for B cell development and maintenance in naive mice. Surprisingly, B cell specific deletion of *Zbtb24* does not evidently compromise germinal center reactions and the resulting primary and secondary antibody responses induced by T cell dependent antigens (TD-Ags), but significantly inhibits T cell independent antigen-elicited antibody productions in vivo. At the cellular level, *Zbtb24*-deficiency specifically impedes the plasma cell differentiation of B1 cells without impairing their survival, activation and proliferation in vitro. Mechanistically, *Zbtb24*-ablation attenuates heme biosynthesis partially through mTORC1 in B1 cells, and addition of exogenous hemin abrogates the differentiation defects of *Zbtb24*-null B1 cells.

**Conclusions:**

*Zbtb24* seems to regulate antibody responses against TD-Ags B cell extrinsically, but it specifically promotes the plasma cell differentiation of B1 cells via heme synthesis in mice. Our study also suggests that defected B1 functions contribute to recurrent infections in patients with ICF2.

**Supplementary Information:**

The online version contains supplementary material available at 10.1186/s11658-024-00641-2.

## Introduction

Immunodeficiency, centromeric instability, and facial anomalies syndrome (ICF [OMIM 242860]) is a rare autosomal recessive and genetically heterogeneous disorder that manifests with multiple system failures [1, 2]. Apart from the hypomethylated pericentromeric regions in specific chromosomes, and varying degrees of facial abnormalities and mental/motor defects, most patients with ICF suffer from recurrent respiratory/gastrointestinal infections in early childhood owing to reduced antibody levels [1, 3]. Approximately 50% of ICF cases (ICF1) have mutations in the DNA methyltransferase 3B (*DNMT3B,* OMIM 602900), and about 30% of patients (ICF2) carry nonsense or missense mutations in the zinc finger and BTB domain containing 24 (*ZBTB24,* OMIM 614064) [3–5]. Although mutations in the cell division cycle associated 7 (*CDCA7*, OMIM 609937), helicase, lymphoid-specific (*HELLS*, OMIM 603946), and ubiquitin-like with plant homeodomain and ring finger domains 1 (*UHRF1*, OMIM 607990) have been recently shown to be associated with ICF3, ICF4, and an atypical ICF case, respectively, there are still few ICF cases (ICFX) left with unidentified disease genes [6, 7]. In line with the similar molecular and clinical characteristics across different ICF subtypes, these disease genes act sequentially/corporately to modulate chromatin accessibilities and DNA methylations [7–13].

*Zbtb24* belongs to a large and evolutionary conserved family of transcriptional regulators with about 60 different members, many of which have prominent roles in hematopoietic development, differentiation and function [14–16]. For instance, *Bcl6* (*Zbtb27*) promotes germinal center (GC) reactions by driving the differentiation of both GC B cells (GCB) and T follicular helper (Tfh) cells, and thereby is indispensable for the generation of high-affinity memory B cells (Bm) and long-lived antibody-secreting plasma cells (PCs) in vivo [14, 16, 17]. In addition, depletion of the ICF4 gene *Hells* in B cell compartment accelerates the premature decay of GCB cells and impairs the generation of high-affinity Bm and antibodies [18]. Thus, the absence of GC structures and circulating memory (but not total) B cells in patients with *ZBTB24*-disrupted ICF2 indicate that ZBTB24 may possess similar functions in antibody responses in vivo [3, 4, 16, 19]. Indeed, ZBTB24 modulates the function of human B lymphoblastoid cell line Raji by indirectly repressing IRF4 and PRDM1 in a BCL6-independent manner [20]. Moreover, ZBTB24 participates in the nonhomologous end joining process, and thereby promotes the class-switch recombination (CSR) in B cells either directly via binding to PARP1 or indirectly by inducing the expression of CDCA7 [21, 22]. Nonetheless, the roles of *Zbtb24* in the development/function of B cells and in vivo antibody responses remain largely unknown.

As the effectors of humoral immunity, B cells are comprised of two populations, the predominant B2 subset and the minor B1 compartment [23]. B2 cells are continuously produced in the bone marrow (BM) from hematopoietic stem cells (HSCs) throughout life, and the newly formed B cells traffic to the spleen where they eventually mature into either follicular B (FOB) or marginal zone B (MZB) cells. By contrast, B1 cells are mainly seeded during fetal and early neonatal life, and the major population is maintained by self-renewal thereafter, although the adult BM may remain limited potential for B1 development [24, 25]. Upon activation by T cell dependent antigens (TD-Ags), the conventional FOB cells enter the GC structure, where they undergo massive proliferation, somatic hypermutation (SHM) and CSR, and ultimately differentiate into Bm or PCs with the capacity to produce high-affinity antibodies [26]. Conversely, MZB and B1 cells are innate-like B cells with limited B cell receptor (BCR) repertoire, and are able to rapidly respond to T cell independent antigens (TID-Ags)/self-antigens by secretion of natural antibodies [24, 25, 27, 28]. They promptly and massively differentiate into mature PCs, and thereby provide antibody-mediated innate immune protection during microbial infections [25, 29]. Given that B1 cells normally reside in the pleura, peritoneum, and intestines, they play an important role in barrier immunity to control microbes at mucosal surfaces. Apart from antimicrobial activities, natural antibodies, most of which are produced by B1 cells, help maintain tissue homeostasis by cross-reacting to epitopes expressed on dead/dying autologous cells [24, 25, 27, 28].

It has been well established that the function and fate of B cells are finely controlled by the proper expressions of different transcription factors (TFs). BCL6/BACH2/PAX5 maintains the phenotype of FOB cells, and promotes their proliferation, SHM and CSR in GC reactions, whereas PRDM1 and XBP1 augment the differentiation of PCs. IRF4 regulates both of these processes, as intermediate IRF4 induces BCL6 and activation-induced cytidine deaminase (AID), which initiates SHM and CSR, while its high expression upregulates PRDM1 [30]. In addition to the timely and spatially regulated expressions of these TFs, recent studies showed that metabolic reprogramming/adaptation shapes the differentiation and function of B cells as well [31–33]. mTORC1 coordinates the increased anabolism and thus plays an essential role in the activation, proliferation and protein synthesis of cells that demand increased energy and building blocks [34]. PKC signaling dictates B cell fate by regulating the mitochondrial biogenesis/function partially through mTORC1 signaling [35]. Coinciding with their instant actions against pathogens, innate-like B1 and MZB cells exhibit augmented glucose/lipid uptake and metabolism as compared to FOB cells [32, 36]. Moreover, high mitochondrial reactive oxygen species (ROS) in activated B cells promotes CSR but dampens the generation of PCs via inhibiting the synthesis of heme, which inactivates BACH2 and thus releases its inhibition on PRDM1 expression and PC differentiation [37, 38].

As the germline deletion of *Zbtb24* is embryonic lethal in mice [8], we herein generated B cell specific *Zbtb24* knockout mice (*Zbtb24*^*B−CKO*^) to investigate its cell-intrinsic role in B cell development and function both in vitro and in vivo. Our data reveal that cell autonomous *Zbtb24* is dispensable for the development/maintenance of B cells in naive mice and the TD-Ag-induced GC reactions and antibody responses, but greatly promotes the PC differentiation of peritoneal B1 cells via augmenting mTORC1 activity and heme biosynthesis upon activation. Thus, our study not only suggests that the barrier defenses against pathogens might be impaired, owing to hampered differentiation of B1 cells, in respiratory and gastrointestinal tracts of patients with ICF2, but also provides mechanistic insights into the specific role of *Zbtb24* in regulating B1 cell function. Our data also indicated that *Zbtb24* may promote antibody responses against TD-Ags, at least in mice, in a B cell and even hematopoietic-cell extrinsic manner.

## Materials and methods

### Mice

**Fig. 1:**
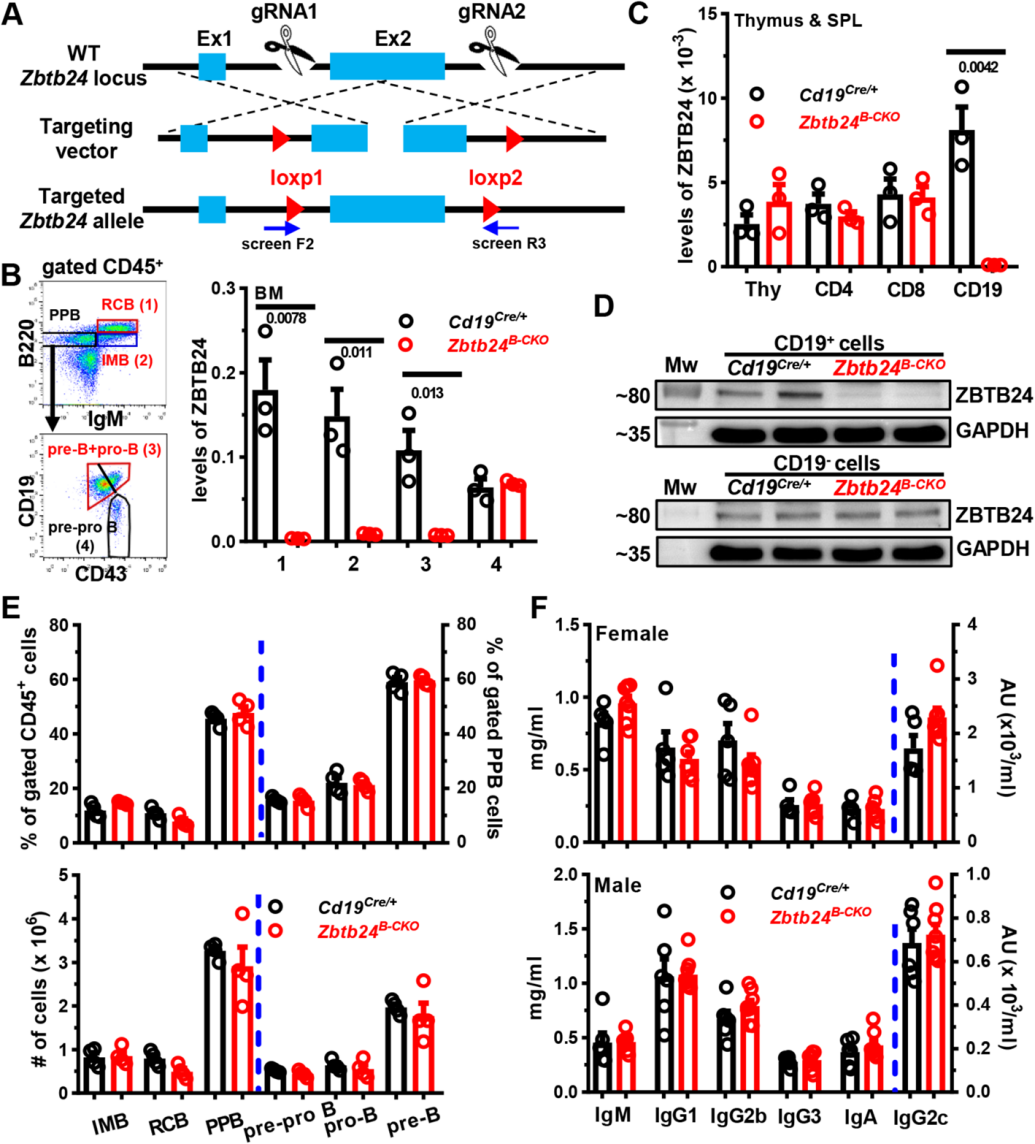
No effect of B cell specific depletion of *Zbtb24* on B cell development in the bone marrow (BM) and baseline serum antibody levels in mice. **A** A schematic representation for generating *Zbtb24*^*loxp/*+^ mice using the CRISPER/CAS9 strategy. Screen F2 and R3 denote primers used to select correctly-targeted ES clones. **B–D** Efficient and specific deletions of ZBTB24 in CD19^+^ B cells in *Cd19*^*Cre/*+^*Zbtb24*^*loxp/loxp*^ (*Zbtb24*^*B−CKO*^) mice. Levels of ZBTB24 were analyzed in FACS-sorted B220^++^IgM^+^ recirculating mature B cells (RCB, gate 1), B220^+^IgM^+^ immature B cells (IMB, gate 2), B220^+^IgM^−^CD19^+^CD43^−^ pre-B plus B220^+^IgM^−^CD19^+^CD43^+^ pro-B cells (gate 3), and B220^+^IgM^−^CD19^−^CD43^+^ pre-pro B cells (gate 4) in the BM (**B**), or FACS-purified CD4^+^ (CD4), CD8^+^ (CD8) T cells and CD19^+^ (CD19) B cells in spleens (SPL) as well as total thymocytes (Thy) (**C**) of control *Cd19*^*Cre/*+^ versus *Zbtb24*^*B−CKO*^ mice by quantitative PCR. Sorting strategies for indicated BM B cell subsets were shown in the left of **B**. Expressions of ZBTB24 were normalized to internal glyceraldehyde 3-phosphate dehydrogenase (GAPDH) (**B**, **C**). Protein levels of ZBTB24 were analyzed in beads-purified splenic CD19^+^ B cells (*upper*) and the remaining CD19^−^ fraction (*lower*) by Western blot (**D**). **E** Bar graphs showing the percents (*upper panel*) or absolute numbers (*lower*) of indicated B cell subsets in the BM of control *Cd19*^*Cre/*+^ and *Zbtb24*^*B−CKO*^ mice. **F** Bar graphs showing levels of total immunoglobulin M (IgM), IgG1, IgG2b, IgG2c, IgG3, and IgA in sera of female (8–10 weeks old) and male (9–11 weeks old) *Cd19*^*Cre/*+^ versus *Zbtb24*^*B−CKO*^ mice. Each symbol/lane represents a single mouse of the indicated genotype, and numbers below horizontal lines indicate *P* values determined by student *t*-test (**B**, **C**). Data in **E**, **F** are representative of two independent experiments. PPB, pre-pro/pro/pre B cells; Mw, molecular weight marker; AU, arbitrary units

The *Zbtb24* conditional allele (*Zbtb24*^*loxp/*+^) was generated using the clustered regularly interspaced short palindromic repeats (CRISPER)/Cas9 strategy (Beijing CasGene Biotech. Co., Ltd). Briefly, C57BL/6 embryonic stem cells (ES) were transfected with the targeting vectors via electroporation. Correctly targeted clones, containing two loxp sites inserted into the flanking regions of *Zbtb24* exon 2 with the translation starting site (Fig. 1A), were identified by polymerase chain reaction (PCR) and injected into blastocytes to generate chimeric mice. Chimeric mice were then crossed with C57BL/6 wild type (WT) mice to obtain F1 mice with the germline-transmitted *Zbtb24*^*loxp/*+^ allele. After multiple-step crossing the heterozygous *Zbtb24*^*loxp/*+^ mouse with *Cd19*^*Cre/*+^ mouse on a C57BL/6 background (kindly provided by Prof. Biao Zheng, East China Normal University), mice with the specific deletion of *Zbtb24* in CD19^+^ B cells (*Zbtb24*^*B−CKO*^, *Cd19*^*Cre/*+^*Zbtb24*^*loxp/loxp*^) were generated. Primers used for ES clone selection and genotyping were listed in Table S1. To exclude the impact of *Cd19*-driven Cre expression on B cells [39], littermate *Cd19*^*Cre/*+^*Zbtb24*^+*/*+^ (*Cd19*^*Cre/*+^) mice were used as controls in all the analysis unless otherwise indicated. Immunodeficient *Rag2*^*−/−*^ C57BL/6 mice with no mature T and B cells (Changzhou Cavens Laboratory Animal Co., Ltd) were used as recipients in adoptive transfer experiments. Mice were housed under specific pathogen-free (SPF) conditions.

### Immunizations

Mice were immunized with the TD-Ag 4-Hydroxy-3-nitrophenylacetyl hapten conjugated to ovalbumin (NP_19_-OVA) emulsified in incomplete Freund adjuvant (IFA, Sigma, 1:1) or adsorbed onto Imject Alum (Thermo Scientific, 1:1), or with the TID-Ag NP-AECM-Ficoll (NP-Ficoll)/NP-LPS intraperitoneally. NP_19_-OVA, NP-Ficoll and NP-LPS were all obtained from Biosearch Technologies.

### B cell isolation and culture

BM cells were harvested by flushing the femurs of mouse, and peritoneal cells were isolated by lavaging the peritoneal cavities (PeC) with 10 ml phosphate buffered saline (PBS). Splenocytes were obtained by mechanically dissociating spleens in PBS, followed by passing through a 70 μM nylon mesh (BD Biosciences). Erythrocytes were removed by addition of ammonium chloride lysis buffer. Total CD19^+^ B, CD19^+^CD23^high^CD21^low^ FOB and CD19^+^CD23^low^CD21^high^ MZB cells in spleens, or peritoneal CD19^+^B220^low^CD23^−^ B1 and CD19^+^B220^high^CD23^+^ B2 cells were sorted out via a FACS Aria cell sorter III (BD Biosciences) with > 95% purity. MojoSort™ Mouse Pan B Cell Isolation Kit II (Biolegend) were used to enrich CD19^+^ B cells in splenocytes before sorting.

Purified B cells were cultured in medium, LPS (L2630, Sigma), goat F(ab’)_2_ anti-mouse IgM (αIgM, SouthernBiotech) or rat anti-mouse CD40 (αCD40, 1C10, Biolegend) in the absence/presence of recombinant mouse IL-4, human TGF-β1 and B cell activating factor (BAFF) (all from Peprotech) in 96 U-bottom plates at 37 °C for indicated times. The complete culture medium was Roswell Park Memorial Institute (RPMI)-1640 (Hyclone) supplemented with non-essential amino acids (Sigma), 10% heat-inactivated fetal bovine serum (FBS, Gibco), 100 U/ml penicillin (Beyotime), 100 μg/ml streptomycin (Beyotime), 50 μM β-mercaptoethanol (Sigma), 1 mM sodium pyruvate, and 10 mM HEPES (Hyclone). In some cases, L-ascorbic acid (L-AC, #A92902, Sigma), hemin (#9039, Sigma), rapamycin (Absin), and corresponding vehicle controls were added at indicated concentrations from the beginning of culture.

### Adoptive transfer experiments

CD4^+^ T cells were isolated with MojoSort™ Mouse CD4 T cell isolation kit (Biolegend) from spleens of WT mice with > 90% purity. Purified splenic CD19^+^ B cells, from *Cd19*^*Cre/*+^ or *Zbtb24*^*B−CKO*^ mice, were mixed with CD4^+^ T cells at the ratio of 2:1 before being injected into *Rag2*^*−/−*^ recipients via the tail vein (5 × 10^6^ B cells plus 2.5 × 10^6^ CD4^+^ T cells/200 μl PBS/mouse). At 1 day later, recipient mice were immunized with NP_19_-OVA emulsified in IFA. To analyze the function of B1 cells in vivo, FACS-sorted peritoneal B1 cells were injected into *Rag2*^*−/−*^ recipients intraperitoneally (2 × 10^5^ cells/200 μl PBS/mouse) followed by NP-LPS immunization.

### ELISA

Mouse sera or B cell culture supernatants were collected at indicated time points and immediately stored at –20 ℃ until the analysis for antibody levels by ELISA. In brief, ELISA plates (Nunc) were coated with goat anti-mouse Ig (SouthernBiotech, 1010–01, 1:1000) or NP_25_-BSA (Biosearch Technologies, 5 μg/ml) to capture all murine Igs or NP-specific antibodies, respectively. After washing with PBS containing 0.05% Tween-20, wells were blocked with PBS containing 3% BSA before incubation with diluted sera or culture supernatants. The optimal dilutions for each Ig subtype were determined by preliminary experiments. Total or NP-specific IgM, IgG1, IgG2b, IgG2c, IgG3, and IgA levels were detected by using HRP-coupled goat anti-mouse subtype-specific secondary antibodies (SouthernBiotech).

### Western blot and Q-PCR analysis

Cells were lysed for 1 h on ice in RIPA lysis buffer (Beyotime) supplemented with protease and phosphatase inhibitors (Selleck). Proteins were separated in an SDS-PAGE 10% gel and subsequently blotted onto a PVDF membrane (Millipore). After incubation with blocking buffer (Tris-buffered saline with 5% nonfat milk), the membrane was incubated with primary antibodies overnight at 4 °C, followed by a further incubation with secondary antibodies at room temperature (RT) for 2 h. Signals were visualized with the Tanon 3500 Gel Imaging System (Tanon). The following antibodies were used: anti-ZBTB24 (PM085, MBL Life Science, 1:1000), phospho-CD19 (Tyr531) antibody (3571 T, CST, 1:1000), anti-GAPDH (AG012, Beyotime, 1:5000), anti-β-ACTIN (T0022, Affinity Bioscience, 1:5000), HRP-coupled donkey anti-Guinea pig (H + L, 706–035-148, Jackson ImmunoResearch Lab), HRP-conjugated goat anti-rabbit IgG (G-21234, Thermo Scientific), and HRP-linked horse anti-mouse IgG (7076P2, CST).

Total RNA was extracted from indicated cells by the Total RNA Kit II (OMEGA). cDNA was subsequently synthesized using Reverse Transcriptase M-MLV (TakaRa) according to manufacturer’s instructions. mRNA levels of indicated genes were quantitatively determined by SYBR-green technology on an ABI-StepOnePlus Sequence Detection System (Applied Biosystems). Sequence of primers used for RT-qPCR analysis were listed in Table S1.

### Flow cytometric analysis

Single cell suspensions were prepared and surface molecules were stained at 4 °C for 30 min with optimal dilutions of each antibody. The following antibodies were used: anti-mouse B220 (RA3-6B2), CD45 (30-F11), CD19 (6D5), CD23 (B3B4), CD5 (53–7.3), CD38 (90), CXCR4 (L276F12), CD69 (H1.2F3), CD86 (PO3), IgG1 (RMG1-1), and IgG2b (RMG2b-1) (all from Biolegend); anti-mouse CD21/35 (eBio4E3 or eBio8D9), CD43 (eBioR2/60), CD93 (AA4.1), and IgM (II/41) (all from eBioscience); and anti-mouse CD138 (281–2), CD95 (Jo2), and IgG3 (R40-82) (all from BD Biosciences). Sometimes 7-AAD (Biolegend) and NP-Ficoll-FITC (NP-FITC, Biosearch Technologies) were additionally used to visualize NP-specific B cells as previously reported [39]. After staining, cells were washed twice with PBS, suspended in 300 μl PBS, and fixed volumes of cells were processed with the Attune^®^ NxT Acoustic Focusing Cytometer (Thermo Scientific). Data were analyzed by FlowJo software (BD Biosciences).

To measure the mitochondrial membrane potential and intracellular ROS, cells were incubated in prewarmed RPMI-1640 containing MitoTracker™ Orange CMTMRos (Thermo Scientific, 50 nM) and DCFH-DA (Beyotime, 5 μM) at 37 °C for 30 min. After extensive washing, cells were further stained with antibodies against surface molecules as indicated above.

For intracellular detection of TFs, cells were fixed/permeabilized with Foxp3 staining buffer set (eBioscience) before incubation with antibodies against BCL6 (7D1), PRDM1 (5E7) and IRF4 (IRF4.3E4) (all from Biolegend). To measure AKT and mTORC1 activity, cells were fixed with 4% paraformaldehyde, permeabilized by ice-cold methanol for 30 min on ice, and then stained with the primary antibody recognizing phosphor-AKT (Ser473) (D9E, CST) or phosphor-S6 ribosomal protein (Ser235/236) (D57.2.E, CST), followed by incubating with the secondary APC-coupled AffinityPure F(ab’)2 fragment donkey anti-rabbit IgG antibody (711–606-152, Jackson Immunolab).

### RNA-sequencing and analysis

Total cellular RNA was extracted with the Total RNA Kit II (OMEGA). Library construction and sequencing was performed on a HiSeq or Novaseq 2 × 150 platform by AZENTA Life Sciences (Suzhou). Sequenced reads were mapped to reference murine genome (mm10 assembly) using bowtie2 with default parameters, and Cufflinks was used to estimate the abundance of transcripts [40, 41]. Quantifications of gene expression were performed using RSEM, and the expression levels of genes in each sample were normalized by means of fragments per kilobase of transcript per million mapped reads (FPKM). These RNA-seq data were deposited in the GEO database with the accession number GSE241746 and GSE241747.

After eliminating absent features (zero counts), differentially expressed genes (DEGs) between two groups with two–three replicates (one mouse per replicate for splenic B cells) were compared via DEseq2. Owing to limited cell numbers post FACS-sorting, PeC B1 cells from two mice of the same genotype were pooled before RNA-extraction and served as one replicate. Genes were considered to be differentially expressed with the cutoff of fold change ≥ 0.414 and *P* value < 0.05. To identify signaling pathways enriched in different samples, we used R package clusterProfiler and software gene set enrichment analysis (GSEA) of MSigDB gene sets (Hallmark, KEGG, and GO) [42, 43]. GSEA was performed for each pairwise comparison using gene lists ranked by the Wald statistic.

### Protoporphyrin IX (PPIX) measurement

Levels of PPIX in cells were analyzed by flow cytometry with excitation at 405 nm and emission at 610/20 nm as described previously [35, 37].

### Statistical analysis

The GraphPad Prism 8.0 software (GraphPad) was used to generate graphs and perform statistical analysis. Data were expressed as mean ± SEM. An unpaired nonparametric Mann–Whitney test was utilized to compare differences among groups unless otherwise indicated. Values at *P* < 0.05 were considered significant.

## Results

### Little effect of *Zbtb24*-deficiency on the development and phenotype of B cells in mice

Given that the germline knockout of *Zbtb24* is embryonic lethal in mice [8], we generated mice harboring a conditional *Zbtb24*^*loxp/*+^ allele with two loxp sites flanking the exon 2, which contains the translation starting site (Fig. 1A). The N-terminal 316 amino acids, including the BTB domain, the AT-hook domain and the first C_2_H_2_ Zinc finger motif, of ZBTB24 protein would be specifically removed in Cre-expressing cells/tissues, which results in a frame shift in the protein coding sequence and thereby generates a premature stop codon. To address cell-intrinsic roles of *Zbtb24* in the development and function of B cells in vivo, we crossed the *Zbtb24*^*loxp/*+^ mice with the *Cd19*-driven Cre-expressing mouse strain (*Cd19*^*Cre/*+^) to generate B cell specific *Zbtb24* deficient (*Zbtb24*^*B−CKO*^, *Cd19*^*Cre/*+^*Zbtb24*^*loxp/loxp*^) and the littermate control (*Cd19*^*Cre/*+^, *Cd19*^*Cre/*+^*Zbtb24*^+*/*+^) mice. *Zbtb24*^*B−CKO*^ mice grew normally, and were phenotypically indistinguishable from the control *Cd19*^*Cre/*+^ mice.

In *Zbtb24*^*B−CKO*^ mice, *Zbtb24* was specifically and efficiently deleted from CD19-expressing pre-B and pro-B cells onward in the BM and periphery, while its expressions in CD19-negative BM pre-pro B cells, splenocytes and thymocytes were intact as analyzed by Q-PCR and/or western blot (Fig. 1B–D). Notably, lack of *Zbtb24* affected neither the percentages/absolute numbers of different developing stages of B cells in the BM (Fig. 1E), nor the numbers of splenic FOB/MZB cells as well as the peritoneal B2, B1a/B1b cells in the periphery (Fig. S1A and B). Moreover, the percentages of GCB in the spleens, mesenteric lymph nodes (MLN) and Peyer’s Patches (PP), and serum total IgM, IgG, and IgA levels were all normal in *Zbtb24*^*B−CKO*^ mice (Fig. S1C and Fig. 1F). Thus, depletion of B cell *Zbtb24* has little impact on the early BM B cell development, the phenotype and maintenance of peripheral B cells, and serum antibody levels in naive mice.

### Largely normal humoral responses to TD-Ags in ***Zbtb24***^***B−CKO***^ mice

**Fig. 2:**
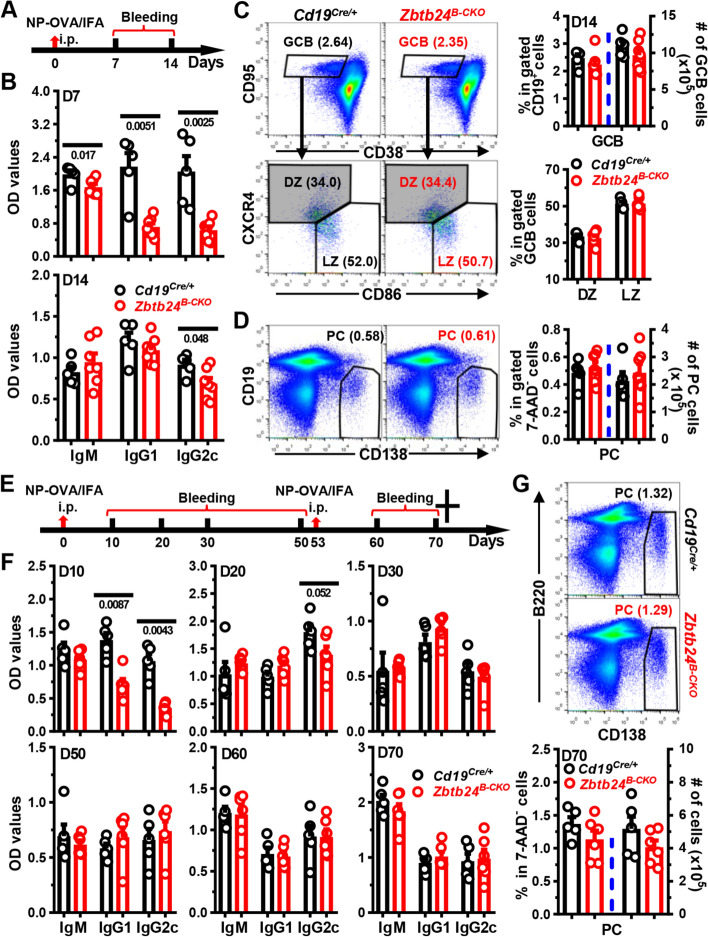
No significant defects in antibody responses in *Zbtb24*^*B−CKO*^ mice post primary/secondary NP-OVA/IFA immunization. Mice (10-week old males in **A-D**; and 8-week old females in **E–G**) were intraperitoneally (i.p.) immunized with NP_19_-OVA emulsified in IFA (1:1, 50 μg/100 μl/mouse) on D0, followed by i.p. rechallenging with NP_19_-OVA/IFA (3:1, 20 μg/100 μl/mouse) on D53 (only in **E**–**G**). Sera were collected on indicated days and NP-specific antibodies were detected by ELISA. Cells in spleens on day (D) 14 (**C**, **D**) or D70 (**G**) were stained with antibodies, in combination with 7-AAD to exclude dead cells, to visualize the percents of CD19^+^CD38^low^CD95^high^ germinal center B (GCB) cells or CD19^−/low^CD138^high^ plasma cells (PC) by flow cytometry. **A**, **E**, schematic diagrams illustrating the immunization and bleeding schedules of mice. **B**, **F**, bar graphs showing the optical density (OD) values of NP-specific antibodies in sera of *Cd19*^*Cre/*+^ and *Zbtb24*^*B−CKO*^ mice on indicated days. **C** representative pseudo-plots showing the percentages of GCB (within gated 7-AAD^−^CD19^+^ B cells) and frequencies of dark zone centroblasts (DZ, CXCR4^high^CD86^low^) and light zone centrocytes (LZ, CXCR4^low^CD86^high^) within gated GCB cells in control *Cd19*^*Cre/*+^ and *Zbtb24*^*B−CKO*^ mice. **D**, **G** Representative pseudo-plots showing the percentages of PCs in spleens of mice on D14 (**D**) or D70 (**G**). Cumulative data on the percentages or absolute numbers of indicated B cell subsets were shown in the right of **C**, **D** or bottom of **G**. Each symbol represents a single mouse of the indicated genotype, and numbers below horizontal lines indicate *P* values while those in brackets denote percentages

The major immunological features of patients with ICF2 are hypogammaglobulinemia and lack of circulating Bm cells, possibly owing to the disrupted germinal center reactions [3, 19, 20, 44]. We thus first characterized TD-Ag-induced humoral responses in mice with B cell specific *Zbtb24* deficiency. Then, 1 week after immunization with NP_19_-OVA emulsified in IFA (NP-OVA/IFA), serum levels of NP-specific IgM, IgG1, and IgG2c were significantly reduced in *Zbtb24*^*B−CKO*^ mice. Surprisingly, on day 14 only NP-specific IgG2c was still slightly decreased, while the other two subtypes were reverted to normal levels in *Zbtb24*^*B−CKO*^ mice (Fig. 2A, B). Moreover, the percentages and numbers of CD19^+^CD95^high^CD38^low^ GCB as well as CD19^low^CD138^++^ PC in spleens were all comparable between control and *Zbtb24*^*B−CKO*^ mice (Fig. 2C, D). Within gated GCB cells, percents of CD86^low^CXCR4^high^ dark zone centroblasts and CD86^high^CXCR4^low^ light zone centrocytes were comparable between the two genotypes of mice as well (Fig. 2C). Consistent with our previous findings in human cells, lack of ZBTB24 did not affect the expressions of BCL6, the key driving TF for GC reactions, in gated either GCB (defined as CD19^+^CD95^high^CD38^low^) compartment or total CD19^+^ B cells (Fig. S2A, B). In addition, the numbers of splenic GCB and PC cells were comparable in *Cd19*^*Cre/*+^ and *Zbtb24*^*B−CKO*^ mice immunized with sheep red blood cells (SRBC) as well (Fig. S2C), further corroborating the dispensability of *Zbtb24* on their differentiations in vivo. Notably, ZBTB24 was efficiently depleted in purified GCB cells (Fig. S2D), thus it is unlikely that ZBTB24-expressing cells outcompete ZBTB24-null ones to fill the GCB compartment in TD-Ag-immunized *Zbtb24*^*B−CKO*^ mice.

Given that the generation of specific antibodies appeared to be delayed in *Zbtb24*^*B−CKO*^ mice upon immunization, we immunized mice with NP-OVA/IFA on day 0, and boosted the mice with the same antigen intraperitoneally once again on day 53 to further determine the impact of B cell *Zbtb24* on humoral responses elicited by TD-Ags in vivo (Fig. 2E). Akin to previous results, control mice produced significantly more NP-specific IgG1 and IgG2c on day 10, and the differences went down greatly on day 20 post the primary immunization (Fig. 2F). Of note, comparable levels of NP-specific antibodies were detected between the two groups thereafter, even on day 7 and 17 post the secondary challenge (Fig. 2F). The numbers of PCs were normal in spleens of *Zbtb24*^*B−CKO*^ as well (Fig. 2G).

We also immunized mice with a high dose of NP_19_-OVA (100 μg/mouse) adsorbed on alum, which predisposes to Th2 immune responses instead of Th1 triggered by IFA in vivo. Again, reduced antigen-specific IgG1 were only observed early (day 7) post the primary immunization (Fig. S3A, B). Moreover, specific deletion of *Zbtb24* in B cell compartment did not affect the ratios of high-affinity to low-affinity (against NP_2_-BSA and NP_25_-BSA, respectively) IgG1 and IgG2c before (i.e., day 70) and two weeks (i.e., day 84) post the secondary immunization (Figure S3C, D). Given that ZBTB proteins may act redundantly to maintain the integrity and function of immune cells [16], we conducted RNA sequencing (RNA-Seq) analysis on purified splenic B cells before and after LPS stimulation to determine if any ZBTB proteins were evidently upregulated to compensate for the loss of *Zbtb24*. Levels of all ZBTB proteins, except ZBTB24, did not differ significantly between time-matched control and *Zbtb24*^*B−CKO*^ splenic B cells (Table S2).

To exclude any potential off-target deletions of *Zbtb24* in *Zbtb24*^*B−CKO*^ mice, we next adoptively transferred purified splenic control or *Zbtb24*-deficient CD19^+^ B cells, together with WT CD4^+^ Th cells, into *Rag2*^*−/−*^ recipients before immunization (Fig. S4A). Although the total IgM and IgG levels were reduced in *Rag2*^*−/−*^ mice reconstituted with *Zbtb24*-depleted B cells, serum levels of antigen-specific IgG, and total numbers of splenic CD19^+^ B cells or CD19^low^CD138^++^ PCs did not differ in *Rag2*^*−/−*^ recipients implanted with control versus *Zbtb24*-deficient B cells (Fig. S4B–D). Decreased total polyreactive IgM might underlie the reduced levels of NP-specific IgM in *Zbtb24*^*B−CKO*^ mice (Fig. S4B, C), as ablation of *Zbtb24* did not profoundly affect the CSR ability of murine splenic B cells in vitro (Fig. S5).

Collectively, these data indicate that B cell intrinsic *Zbtb24* does not play an essential role in the generation and maintenance of GCB cells, their affinity maturation as well as later differentiation toward Bm or PCs after immunization, albeit that *Zbtb24*^*B−CKO*^ mice show delayed/mitigated early antibody productions induced by TD-Ag. Notably, mice with a specific depletion of *Zbtb24* in T cells (*Cd4-Cre*) or the whole hematopoietic lineages (*Vav1-Cre*) exhibit largely intact antibody responses in vivo before and after TD-Ag immunizations (Fig. S6A–D), implying that *Zbtb24* may modulate TD-Ag-elicited GC reactions and antibody responses outside of the hematopoietic system.

### Significantly reduced humoral responses to TID-Ags in ***Zbtb24***^***B−CKO***^ mice

**Fig. 3:**
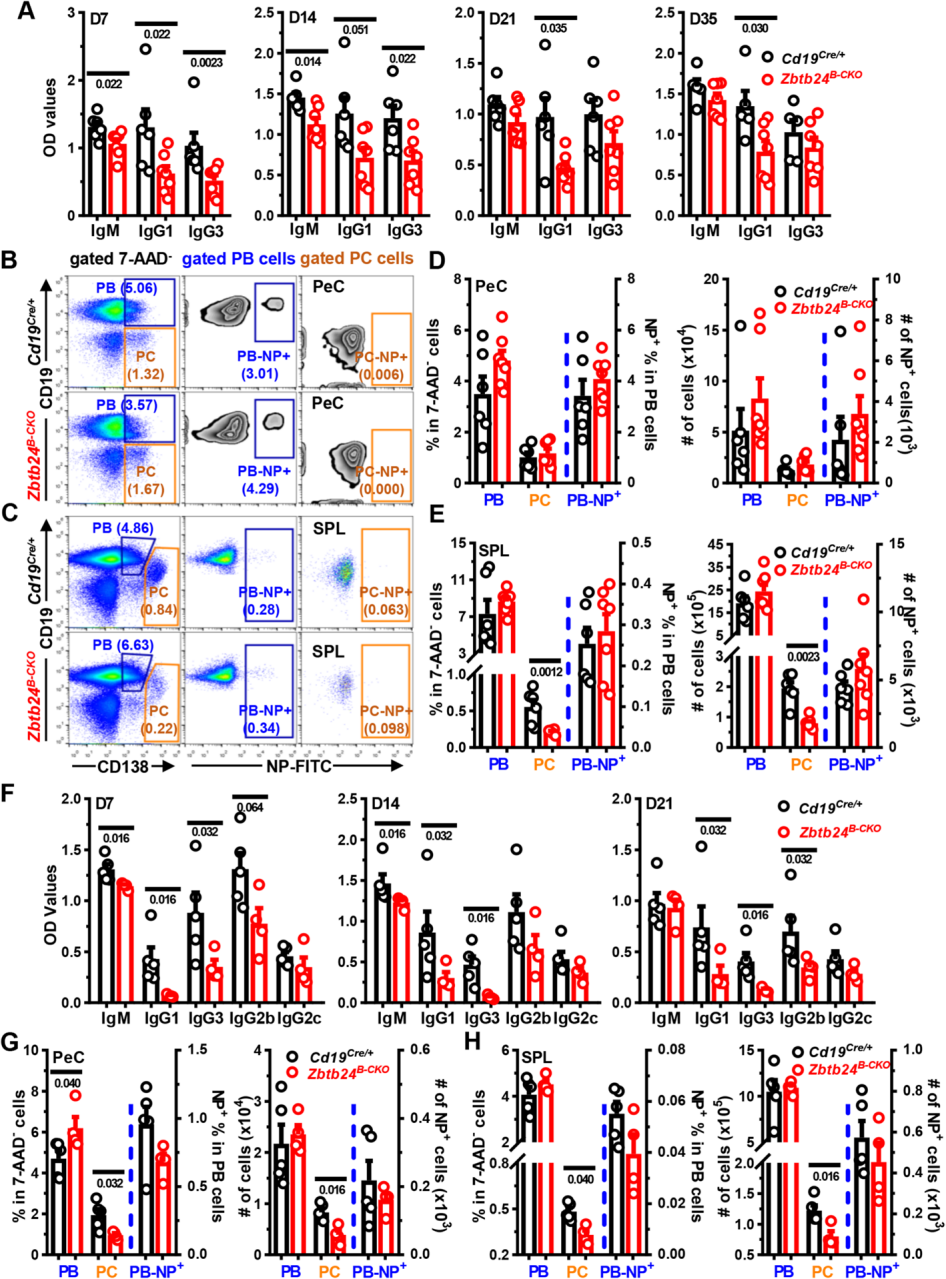
Significantly reduced antigen-specific antibody levels in *Zbtb24*^*B−CKO*^ mice after TID-Ag immunization. Mice were intraperitoneally immunized with the type II TID-Ag NP-Ficoll (10 μg/200 μl PBS/mouse, **A**–**E**) or type I TID-Ag NP-LPS (20 μg/200 μl PBS/mouse, **F**–**H**) on D0. Blood was taken at indicated times and NP-specific antibodies in sera were determined by ELISA in plates coated with NP_25_-BSA. **A**, **F** Bar graphs showing the optical density (OD) values of NP-specific antibodies in diluted sera of *Cd19*^*Cre/*+^ versus *Zbtb24*^*B−CKO*^ mice on indicated days post immunization with NP-Ficoll (**A**) or NP-LPS (**F**). **B**, **C** Representative pseudo/zebra-plots illustrating the gating strategies for CD19^+^CD138^+^ plasma blasts (PB)/CD19^low^CD138^high^ plasma cells (PC) and percents of NP^+^ cells within gated PB (PB-NP^+^) and PC (PC-NP^+^) cells in peritoneal cavities (PeC, **B**) or spleens (SPL, **C**) of mice on D35 post NP-Ficoll immunization. **D**, **E**, **G**, **H**, bar graphs showing the percentages and absolute numbers of PB and PC cells as well as the NP^+^ cells within gated PB/PC cells (PB-NP^+^/PC-NP^+^, respectively) in PeC (**D**, **G**) and SPL (**E**, **H**) of mice on D35 post NP-Ficoll (**D**, **E**) or on D21 post NP-LPS (**G**, **H**) immunization. Each symbol represents a single mouse of the indicated genotype (female, 10 weeks of age in **A**–**E**; and male, 9 weeks of age in **F**–**H**), and numbers below horizonal lines in bar graphs indicate *P* values. Data in **A**, **F** are representative of two independent experiments

The reduced total serum IgM and IgG in *Rag2*^*−/−*^ mice reconstituted with *Zbtb24*-deficient total splenic B cells, most of which likely react against autoantigens and commensal gut bacteria, indicate that lack of *Zbtb24* hampers antibody responses elicited by TID-Ags (Figure S4B). Indeed, *Zbtb24*^*B−CKO*^ mice produced significantly decreased amounts of NP-specific IgM, IgG1, and IgG3, three major antibody subtypes against soluble protein or carbohydrate antigens in mice [39], in the first 2 weeks after immunization with the type II TID-Ag NP-Ficoll. On day 21 and 35, levels of IgG1 were still reduced, and the other two antibody subtypes tended to decrease as well (Fig. 3A). Likewise, significantly less NP-specific IgM, IgG1, IgG3, and IgG2b were detected in *Zbtb24*^*B−CKO*^ mice after immunization with the type I TID-Ag NP-LPS (Fig. 3F). As expected, reduced percentages/numbers of CD19^low^CD138^++^ PCs were detected in spleens and/or peritoneal cavities (PeC) of *Zbtb24*^*B−CKO*^ mice (Fig. 3B–E and G–H). Moreover, depletion of *Zbtb24* in hematopoietic cells resulted in diminished antibody productions induced NP-Ficoll as well (Fig. S6E). These data strongly indicate that B cell intrinsic *Zbtb24* promotes the differentiation of PC and production of antibodies against TID-Ags in vivo.

B1 and MZB cells are considered the main responders to TID-Ags [45]. We thus tried to delineate which cell type was responsible for the diminished antibody levels in TID-Ag-immunized Zbt24^B−CKO^ mice via detailed flow cytometry analyses. At 5 weeks post NP-Ficoll immunization, the numbers of NP-specific cells in gated different B cell compartments were all comparable in the spleens and PeC between *Cd19*^*Cre/*+^ and *Zbtb24*^*B−CKO*^ mice (Fig. S7), possibly owing to the late analyzing time point. Of note, significantly less NP^+^ B1, but not FOB or MZB, cells were observed in the spleens of *Zbtb24*^*B−CKO*^ mice 3 weeks post NP-LPS inoculation, and NP^+^ B1 cells tended to decrease in their PeC as well (Fig. S8), indicating that B1 was the target B cell subset through which *Zbtb24* promoted TID-Ag-induced antibody responses in vivo.

### *Zbtb24*-deficiency impairs the differentiation and antibody-producing ability of B1 cells in vivo

**Fig. 4:**
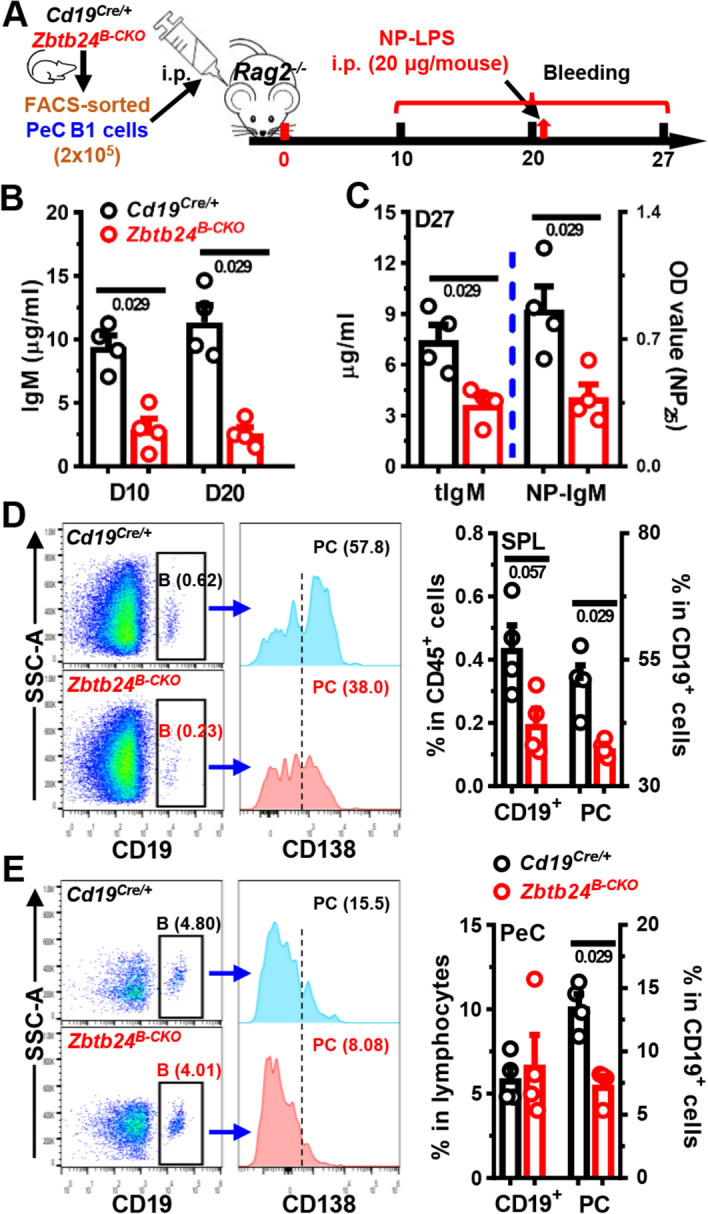
Impaired function of *Zbtb24*-deficient B1 cells in vivo. FACS-sorted peritoneal cavity CD19^+^B220^low^CD23^−^ B1 cells from control *Cd19*^*Cre/*+^ and *Zbtb24*^*B−CKO*^ mice (female, 12 weeks of age) were adoptively transferred into *Rag2*^*−/−*^ recipient mice (female, 8 weeks of age) intraperitoneally (2 × 10^5^ cells/200 μl PBS/mouse). On D20 post injection, *Rag2*^*−/−*^ recipient mice were immunized with NP-LPS (20 μg/200 μl PBS/mouse) intraperitoneally. Blood was taken at indicated times and cells in peritoneal cavities (PeC) and spleens (SPL) were analyzed by flow cytometry on D27. **A** A schematic flow-chart showing the experiment setup. **B**, **C** Bar graphs showing levels of total (**B** and tIgM in **C**) and NP-specific (NP-IgM in **C**) IgM in sera of recipient mice on D10 and D20 (**B**) or D27 (**C**). **D**, **E** Representative offset histograms/bar graphs showing the percentages of CD19^+^ B cells and CD19^+^CD138^+^ plasma cells (PC) within gated B cells in the spleens (SPL, **D**) and peritoneal cavities (PeC, **E**) of recipients implanted with *Cd19*^*Cre/*+^ or *Zbtb24*^*B−CKO*^ B1 cells. Each dot represents a single recipient mouse, and numbers below horizontal lines indicate *P* values. Data are representative of two experiments

To unambiguously reveal the roles of *Zbtb24* in B1 cells in vivo, we adoptively transferred peritoneal B1 cells, purified from *Cd19*^*Cre/*+^ or *Zbtb24*^*B−CKO*^ mice by FACS-sorting, into *Rag2*^*−/−*^ mice with no mature T/B cells and serum antibodies (Fig. 4A). Total IgM levels were reduced by ~ 70% in *Rag2*^*−/−*^ recipients received *Zbtb24*-deficient B1 cells (Fig. 4B). Moreover, 7 days after NP-LPS immunization (i.e., day 27 post transfer), *Zbtb24*-deficient B1-reconstituted *Rag2*^*−/−*^ recipients produced significantly less NP-specific IgM than those harboring control *Cd19*^*Cre/*+^ B1 cells (Fig. 4C). Total or NP-specific IgG was barely detectable in recipients implanted with *Zbtb24*-deficient or sufficient B1 cells (data not shown).

Upon activation, some peritoneal B1 cells transit to the spleen to divide and differentiate into PCs, while others stay in PeC and rapidly differentiate into PCs even without division [46]. We thus compared the number of B and PCs in the spleens and PeC of recipients. The percentages of *Zbtb24*-deficient CD19^+^ B cells and CD138^+^ PCs were significantly reduced in spleens, while only the latter was decreased in the PeC of recipients (Fig. 4D, E).

In sum, our data (depicted in Figs. 3, 4, and S6E, S7, S8) indicate that lack of ZBTB24 protein suppresses the differentiation of B1 cells toward PCs and thereby reduces the antibody levels against TID-Ags in vivo. Given that surface levels of CD11b, which couples with CD18 to form MAC-1/CR3 and governs the migration of B1 cells from the PeC into the spleens [46, 47], and surface IgM were comparable on *Zbtb24*-deficient and -sufficient peritoneal B1 cells (Fig. S9A, B), it is unlikely that reduced PC differentiation of B1 cells was attributable to impaired migration or BCR-triggering.

### *Zbtb24*-deficiency impedes the PC differentiation of B1 cells without compromising their survival, activation and proliferation in vitro

**Fig. 5:**
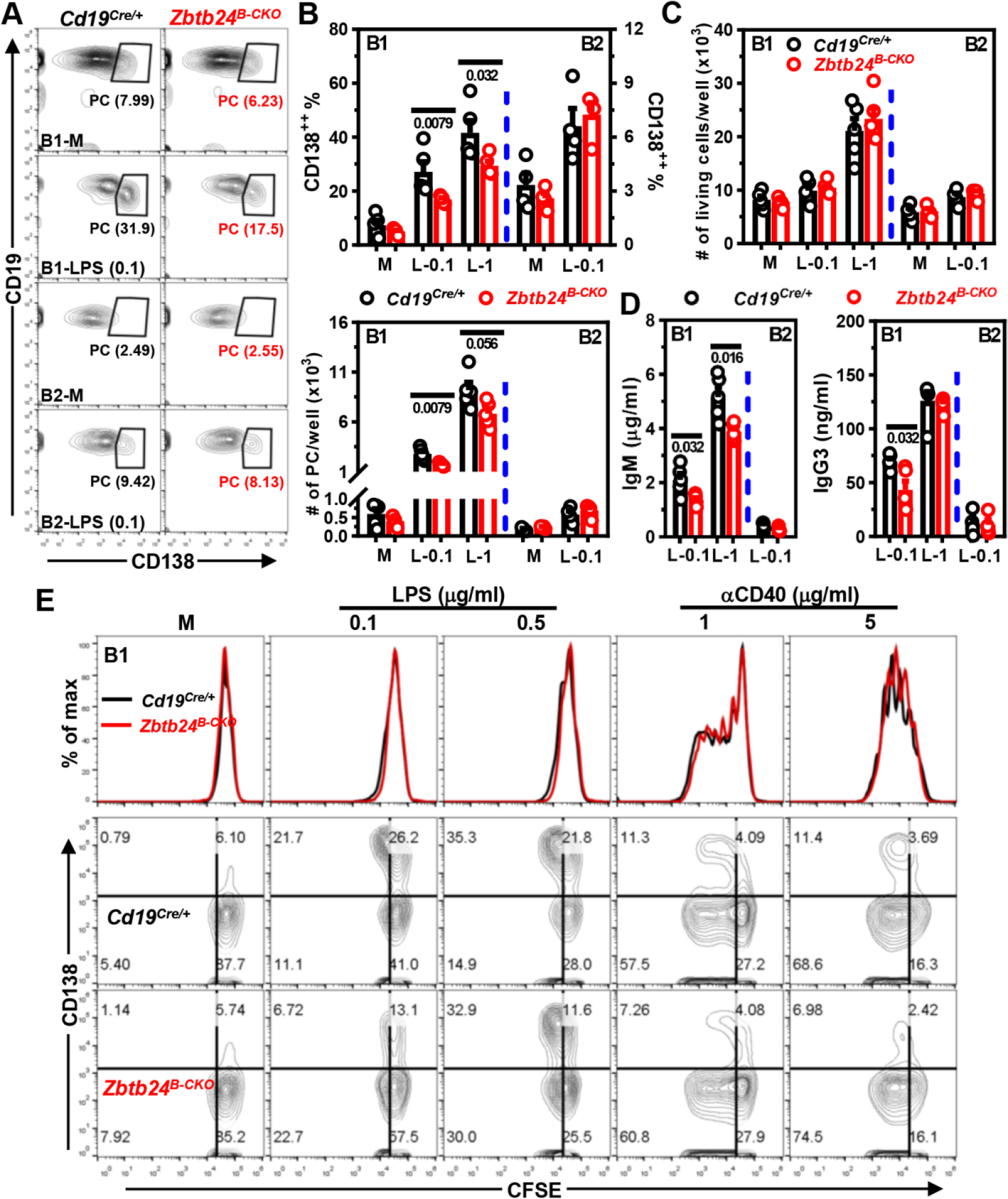
*Zbtb24*-deficiency specifically inhibits LPS-induced differentiation of peritoneal B1 cells toward PCs in vitro. CD19^+^B220^low^CD23^−^ B1 and CD19^+^B220^high^CD23^+^ B2 cells were FACS-sorted from peritoneal cavities of *Cd19*^*Cre/*+^ and *Zbtb24*^*B−CKO*^ mice, and subsequently cultured (2–5 × 10^4^ cells/well in 96-U bottom plate) in medium (M), 0.1/1 μg/ml LPS (L-0.1/L-1, respectively) for 3 days. **A** Representative contour-plots showing the percentages of CD19^low^CD138^+^ PCs in differently cultured B1 or B2 cells on day 3. **B**, **C** Bar graphs showing the percentages/absolute numbers of PCs (**B**) or total living cells (**C**) 3 days post stimulation. **D** Bar graphs showing the IgM and IgG3 levels in culture supernatants of LPS-stimulated B1 or B2 cells on day 3. Antibody levels in supernatants of B cells cultured in medium were too low to be detected after 5 × dilution. Each dot represents a single mouse of the indicated genotype (male, 10 weeks of age). **E** Representative overlayed histograms showing the comparable division profiles (top panel) or contour-plots showing the proliferation (CFSE) versus differentiation (CD138, middle and bottom panels) of cultured peritoneal B1 cells isolated from *Cd19*^*Cre/*+^ versus *Zbtb24*^*B−CKO*^ mice (male, 14 weeks old, *n* = 3 and 4 for *Cd19*^*Cre/*+^ and *Zbtb24*^*B−CKO*^, respectively). B1 cells were labeled with the cell division tracker CFSE (10 μM) and subsequently cultured in medium (M), LPS (0.1/0.5 μg/ml) or anti-IgM (αCD40, 1/5 μg/ml) for 3 days before flow cytometry analysis. Numbers below horizontal lines in **B**–**D** indicate *P* values. Data are representative of 2–4 independent experiments

Upon activation by LPS in vitro, the differentiation of purified peritoneal B1, but not B2, cells towards CD138^+^ PCs was significantly impaired by *Zbtb24* depletion (Fig. 5A, B). Accordingly, levels of IgM and IgG3 in culture supernatants were reduced in LPS-cultivated *Zbtb24*-null B1 cells (Fig. 5D). Given that B1 cells are more sensitive to TLR agonists-induced PC differentiation than FOB cells [29], we further cultured splenic B cells with anti-IgM/CD40 or high amounts of LPS to mimic the T-cell dependent or independent stimulations, respectively. Again, deficiency of *Zbtb24* did not suppress the PC differentiation of splenic B2 cells as well as their antibody producing abilities in these conditions (Fig. S9C–E), corroborating our in vivo findings depicted in Figs. 2D, G and S4D. *Zbtb24*-deficiency did not reduce the viabilities and cell numbers in these B1/B2 cultures (Figs. 5C, and S10A, D, E). Moreover, the ability of B1/B2 cells to upregulate surface activation makers (CD69 and CD86) were not impaired by *Zbtb24* depletion (Fig. S10B, C, F), excluding a generally disrupted signaling transduction downstream of TLR4 in B cells deprived of *Zbtb24*.

As reported previously [48], B1 cells were anergic to BCR triggering and thus failed to undergo activation upon anti-IgM stimulation in vitro (Fig. S10B, C, F). To further determine the role of ZBTB24 in cell proliferation, we labeled B1 cells with cell division tracker CFSE before culture. Interestingly, LPS stimulation induced massive PC differentiation accompanied by minimal cell divisions, whereas anti-CD40 elicited robust proliferation concurrent with little CD138 upregulation (Fig. 5E), implying that B1 cells were able to uncouple PC differentiation from the cell division process. Of note, *Zbtb24*-depletion significantly repressed PC differentiation of B1 cells without impeding their mitoses induced by LPS or anti-CD40 in vitro (Fig. 5E).

On the basis of these data, we conclude that ZBTB24 promotes the PC differentiation of B1 cells independent of their survival, activation and proliferation in vitro.

### *Zbtb24*-deficiency inhibits the PC differentiation of B1 cells via attenuating heme synthesis

**Fig. 6:**
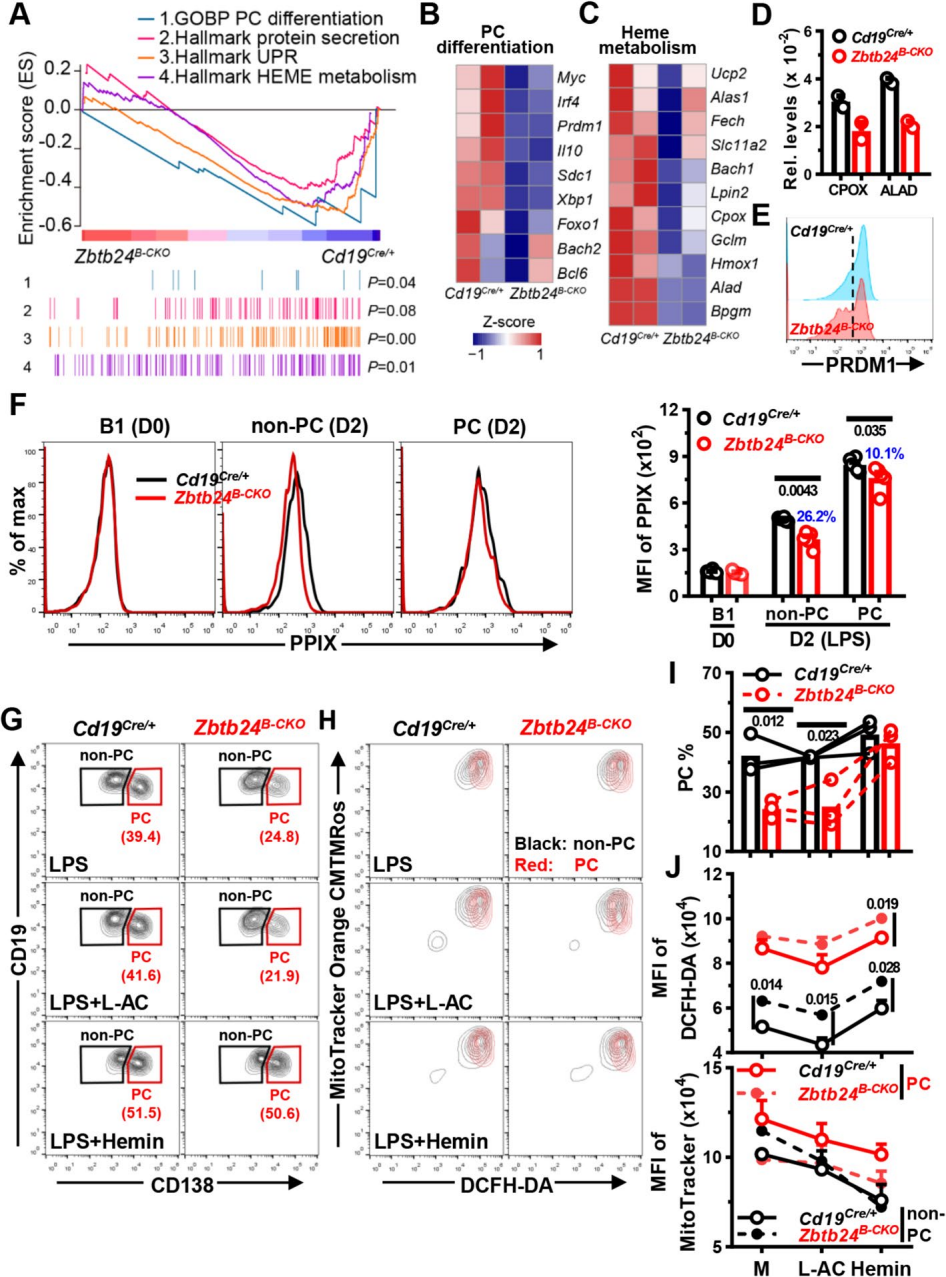
*Zbtb24*-deficiency blunts the differentiation of B1 cells into PCs through repressing the biosynthesis of heme. FACS-sorted peritoneal CD19^+^B220^low^CD23^−^ B1 cells were stimulated with 0.1 μg/ml LPS in 96-U bottom plate for 24 h (RNA-seq/Q-PCR, **A-D)** or 2–3 days without/with additional L-AC (250 μM)/Hemin (25 μM) to induce PC differentiation (**E**–**J**). **A**, GSEA plots of genes involved in PC differentiation, protein secretion, unfolded protein response (UPR), and heme metabolism in LPS-stimulated *Zbtb24*^*B−CKO*^ versus * Cd19*^*Cre/*+^ B1 cells. **B**, **C**, heatmaps showing the *z*-score normalized on the raw expression counts of dysregulated genes regulating the differentiation of PC cells (**B**) or heme metabolism in cells (**C**) identified by RNA-seq analysis. The complete lists of enriched genes regulating UPR and heme metabolism were provided in Fig. S11C. **D** mRNA levels of CPOX and ALAD in *Cd19*^*Cre/*+^ versus *Zbtb24*^*B−CKO*^ B1 cells determined by Q-PCR. **E** Representative half-offset histograms showing the levels of intracellular PRDM1 in LPS-stimulated B1 cells on D2. **F** Representative overlayed histograms (left) and cumulative data (right) showing the levels of intracellular PPIX in resting control versus *Zbtb24*^*B−CKO*^ B1 cells (D0) or gated CD138^−^ non-PC and CD138^+^ PC cells in LPS-stimulated cultures on D2. **G**, **I** Representative contour-plots (**G**) and bar graphs (**I**) showing the percentages of CD19^low^CD138^+^ PCs in differentially cultured *Cd19*^*Cre/*+^ versus *Zbtb24*^*B−CKO*^ B1 cells on D3. **H** Representative overlayed contour-plots showing the intracellular ROS levels (visualized by DCFH-DA) and mitochondrial mass/membrane potentials (detected by MitoTracker Orange CMTMRos) in gated CD138^−^ non-PC (black) versus CD138^+^ PC (red) cells in cultured B1 cells on D3. **J** Bar graphs showing the ROS levels (MFI of DCFH-DA, top) or mitochondrial mass/membrane potential (MFI of MitoTracker, bottom) in gated non-PC/PC cells in *Cd19*^*Cre/*+^ versus *Zbtb24*^*B−CKO*^ B1 cultures. Gates for CD138^−^ non-PC and CD138^+^ PC were illustrated in **G**. Each dot represents a single mouse of the indicated genotype in **D**, **F** and **I** (male, 12–14 weeks of age), while results in **J** are expressed as mean ± SEM (*n* = 3). Numbers in **F**, **I** and **J** indicate *P* values determined by student *t*-test. Blue numbers in **F** denote percents of reduction. Data in **E**–**J** are representative of two experiments

We next performed RNA-Seq analysis to explore how ZBTB24 regulates the differentiation of B1 cells. Upon activation by LPS, *Zbtb24*-deficient peritoneal B1, but not splenic B2 cells, expressed significantly lower levels of hallmark genes involved in PC differentiation, unfolded protein response (UPR), and protein secretion, such as *Prdm1* (the driving force of PC differentiation), *Sdc1* (encoding CD138), *Il10*, *Irf4*, and *Xbp1* (Fig. 6A, B, E, and Table S2). Of note, expressions of *Bach2* and *Bcl6*, two major TFs repressing *Prdm1*, were not influenced by *Zbtb24* depletion in B1 cells (Fig. 6B). Therefore, it is unlikely that the B1-specific effect of ZBTB24 on PC differentiation is attributable to its direct binding and regulation of these key regulators.

Enormous studies recently showed that the activation and function of lymphocytes are intertwined with metabolic reprogramming and adaption [31–33]. In association with the altered function, multiple genes relating to nutrients uptake and metabolism, such as *Slc2a1/Glut1*, *Slc16a3/Mct3*, *Ak4*, *Pdk1*, *Pfkl*, and *Tpi1*, were significantly upregulated in *Zbtb24*^*B−CKO*^ B1 cells (Fig. S11A). Accordingly, GSEA identified a positive enrichment of gene sets regulating the glycolysis and pyruvate metabolism in *Zbtb24*-null B1 cells (Fig. S11B). By contrast, a panel of genes involved in heme metabolism were downregulated in *Zbtb24*^*B−CKO*^ cells, albeit that the differences did not reach the cutoff value and thus were much milder compared with *Zbtb24* direct targets, such as *Cdca7* and *Ostc* (Figs. 6A, C, and S11C). Expressions of *Alad* (5-aminolevulinic acid dehydratase) and *Cpox* (Coproporphyrinogen oxidase), two enzymes that directly catalyze reactions along the heme synthesis pathway in cytosol and mitochondrial intermembrane space, respectively [49], were consistently reduced in *Zbtb24*-null B1 but not B2 cells (Fig. 6C, D, S11C, and Table S2). Moreover, the level of *Hmox1*, a heme-induced oxygenase that mediates its degradation and serves as a readout for intracellular heme content [38], was mildly decreased in *Zbtb24*^*B−CKO*^ B1 cells as well (Fig. 6C). Given that heme promotes PRDM1 expression and PC differentiation of B cells by inactivating BACH2 [38], we reasoned that heme biosynthesis may be attenuated in *Zbtb24*-depleted B1 cells, thereby leading to impaired PC differentiation.

Indeed, *Zbtb24*-depletion significantly mitigated LPS-induced upregulation of PPIX, the final substrate of heme biosynthesis [49], in B1 cells, albeit no differences were observed in resting cells (Fig. 6F). Notably, ablation of *Zbtb24* resulted in a more pronounced PPIX reduction in CD138^−^ non-PC cells than that in CD138^+^ PC cells (26.2% versus 10.1%, Fig. 6F). Moreover, addition of exogenous hemin in cultures almost completely abrogated the differentiation defects of *Zbtb24*-deficient B1 cells (Fig. 6G, I), while its supplementation similarly promoted/suppressed PC differentiation/CSR in control versus *Zbtb24*^*B−CKO*^ splenic B cells (Fig. S12A, B). Together, these data indicate that ZBTB24 specifically promotes the PC differentiation of B1 cells via augmenting heme synthesis. Exogenous hemin only slightly potentiated the PC differentiation of control B1 cells (Fig. 6G, I), implying that activated peritoneal B1 cells may contain abundant intracellular heme to facilitate their accelerated and heightened PC differentiation.

### *Zbtb24*-depletion represses the heme synthesis partially through mTORC1 in B1 cells

The rather mildly downregulated genes along heme synthesis pathway, compared with *Zbtb24*-direct targets, such as *Cdca7*, implied that ZBTB24 exerts the modulation of former genes indirectly. It has been recently shown that endogenous ROS inhibits heme synthesis, and activated splenic B cells with intermediate mitochondrial mass/membrane potential are predisposed to become PCs [37]. We thus wondered whether ZBTB24 promoted heme synthesis in B1 cells via regulating intracellular ROS and/or mitochondrial function. Compared with activated splenic B cells, B1 cells had significantly higher levels of ROS, which were further increased by *Zbtb24*-depletion (Figs. 6H, J, and S12C, D). However, addition of the antioxidant L-ascorbic acid (L-AC) failed to promote PC differentiation in peritoneal B1 cells, albeit that it did reduce ROS levels in B1 cell and augment the PC differentiation of splenic B2 cells (Figs. 6G–J, and S12). The mitochondrial mass/membrane potential was markedly lower in splenic B2-derived PCs as previously reported [37], but no such differences were observed in B1 cultures, and no significant impact of *Zbtb24*-deficiency was observed (Figs. 6H, J, and S12C, D). Thus, the metabolic reprogramming and mitochondrial function differ significantly between B1 versus B2 cells along their differentiation toward PCs. Because ROS represses the final step of heme synthesis by inhibiting the addition of ferrous ions to PPIX [37], it is unlikely that disturbed ROS levels were responsible for the attenuated PPIX accumulation in *Zbtb24*-deficient B1 cells.

**Fig. 7:**
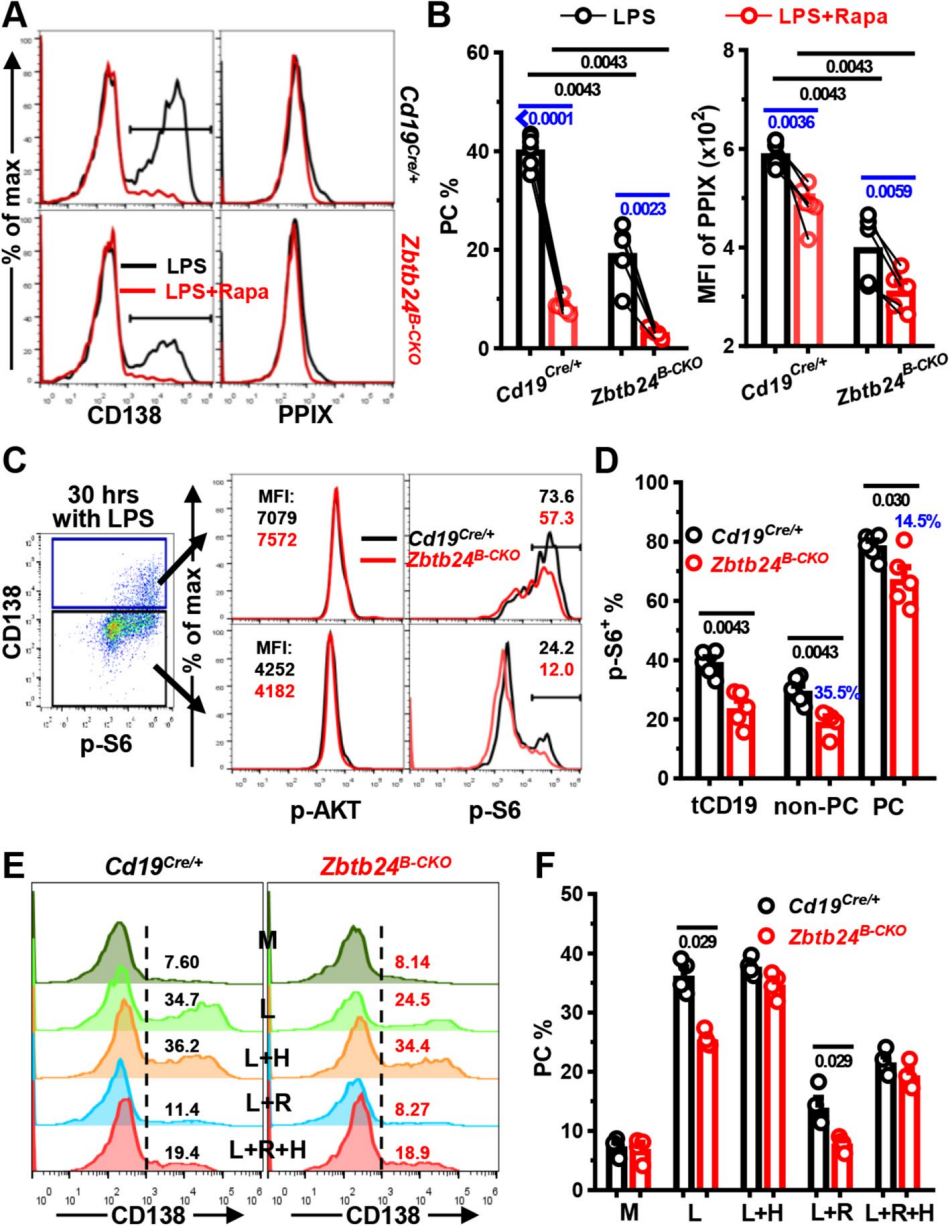
*Zbtb24*-ablation represses the mTORC1 activity, which promotes heme synthesis and PC differentiation of B1 cells. Peritoneal CD19^+^B220^low^CD23^−^ B1 cells were stimulated with 0.1 μg/ml LPS in the absence/presence of rapamycin (Rapa, 10 nM) for ~ 2 days (**A**–**D**). **A**, **B** Representative overlayed histograms (**A**) and bar graphs (**B**) showing the PC differentiation (left panel) and PPIX expression (right panel) in B1 cells cultured with LPS ± Rapa on day 2. **C**, **D** representative FACS-plots/overlayed histograms (**C**) and bar graphs (**D**) showing the expression of phosphorylated AKT (p-AKT) or ribosomal protein S6 (p-S6) in gated CD138^+^ PC or CD138^−^ non-PC cells derived from *Cd19*^*Cre/*+^/*Zbtb24*^*B−CKO*^ B1 cells after stimulation with LPS for 30 h. tCD19 denotes total CD19^+^ B cells and blue numbers in **D** indicate percent of reduction in gated *Zbtb24*^*B−CKO*^ populations compared with the corresponding control counterparts. **E**, **F** Representative histograms (**E**) and bar graphs (**F**) showing the PC differentiation of B1 cells cultured in medium (M), LPS (L), and LPS plus Hemin (25 μM, L + H), rapamycin (10 nM, L + R) or both (L + R + H) for 2 days. Each dot represents a single mouse of the indicated genotype (12-week males in **A**–**D**, and 9-week females in **E**, **F**). Black/blue numbers below horizontal lines indicate *P* values determined by Mann–Whitney test or paired *t*-test, respectively. Data are representative of two experiments

Expressions of *Slc3a2* and *Slc7a5*, encoding the respective heavy and light chain of CD98, positively correlate with mTORC1 activity in B cells [35]. RNA-Seq data showed that levels of *Slc3a2* and *Slc7a5* were decreased, coinciding with the disturbed mTORC1 signaling in activated *Zbtb24*-null B1 cells as revealed by GSEA (Fig. S11A, B). mTORC1 activity regulates the protein synthesis and metabolism of mammalian cells, and promotes PC differentiation of murine splenic B cells partially via augmenting heme synthesis [35]. In keeping with these findings obtained in B2 cells, inclusion of an mTORC1 specific antagonist, rapamycin, significantly suppressed the PC differentiation and PPIX accumulation in peritoneal B1 cells (Fig. 7A, B), demonstrating that mTORC1 promotes heme synthesis and PC differentiation of B1 cells as well. In line with this notion, *Zbtb24*-depletion significantly repressed the expression of phosphorylated ribosomal protein S6 (p-S6), an event controlled by mTORC1 activity [35], in LPS-stimulated B1 cells, albeit that no differences were observed in the upstream AKT activity (Fig. 7C, D). Notably, the inhibition of p-S6 was more pronounced, akin to PPIX contents, in CD138^−^ cells as compared with that in CD138^+^ PCs (Figs. 6F, and 7C, D), indicating that the impaired mTORC1 activity underlies, at least partially, the attenuated heme synthesis and PC differentiation of *Zbtb24*-null B1 cells.

We noticed that PC differentiation and intracellular PPIX levels were still considerably reduced in mTORC1 repressed (i.e., cultured with LPS plus rapamycin) *Zbtb24*-null B1 cells (Fig. 7A, B). To determine the extent to which the two factors (mTORC1 activity versus heme accumulation) contribute to the defected PC generation of *Zbtb24*-deficient B1 cells, we differentiated B1 cells in the presence of rapamycin/hemin alone or both (Fig. 7E, F). Supplementation of hemin not only completely reverted the defects caused by *Zbtb24*-depletion as previously observed in Fig. 6G, I, but also partially relieved the inhibitory effect of rapamycin on PC differentiation of B1 cells. Of note, control and *Zbtb24*-null B1 cells exhibited comparable PC differentiation in the presence of both rapamycin and hemin (Fig. 7E, F), implying that attenuated intracellular heme synthesis is mainly responsible for the differentiation defects of *Zbtb24*-deficient B1 cells.

Collectively, our data show that ZBTB24 promotes the PC differentiation of B1 cells mainly through augmenting heme synthesis, and the attenuated heme biosynthesis in *Zbtb24*-depleted B1 cells is partially attributed to their impaired mTORC1 activity.

### No effect of *Zbtb24*-deficiency on PC differentiation of MZB cells

**Fig. 8:**
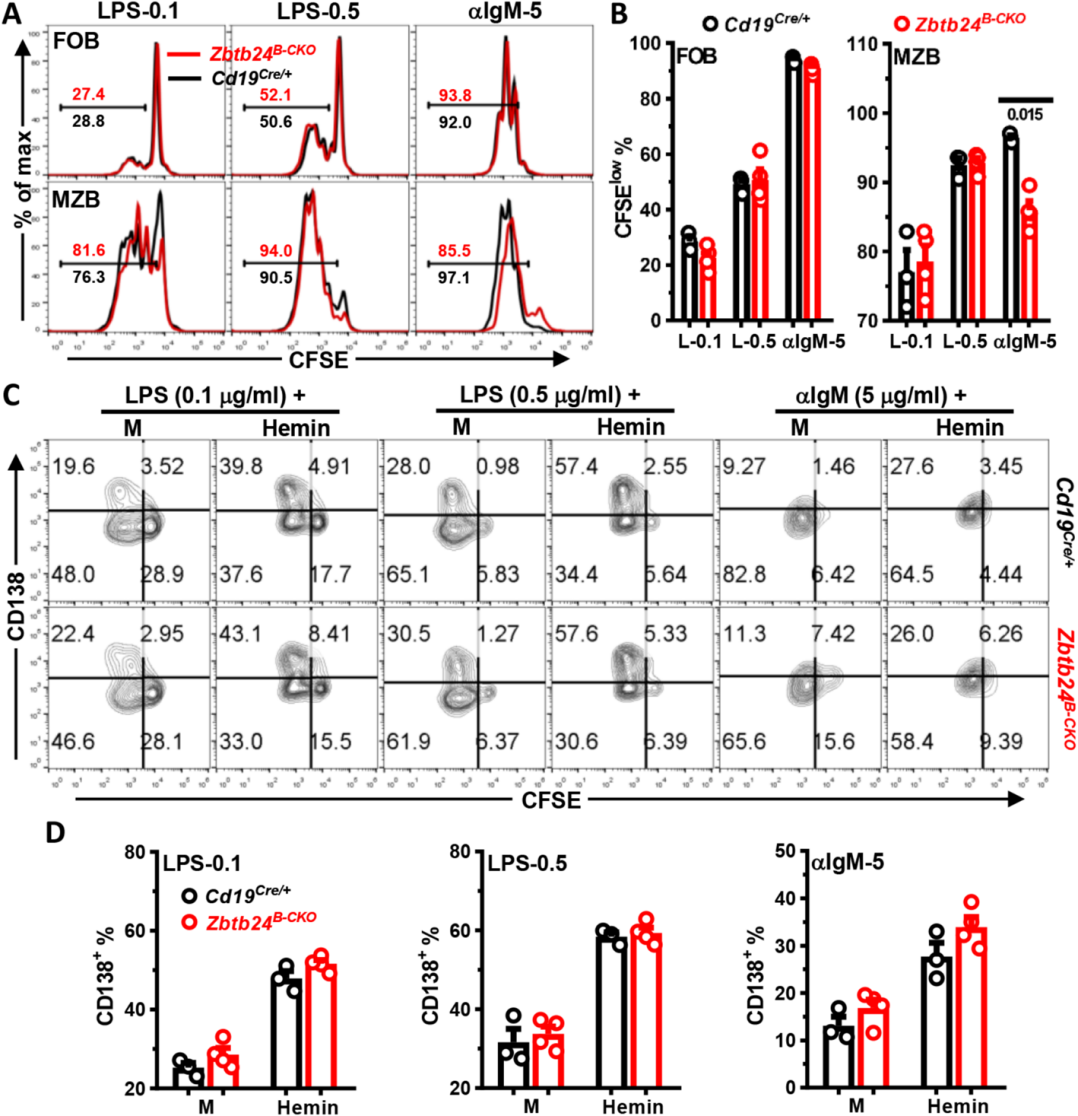
*Zbtb24*-deficiency has no effect on the differentiation, but slightly reduces BCR-triggered proliferation of MZB cells in vitro. CD19^+^CD23^high^CD21^low^ FOB and CD19^+^CD23^low^CD21^high^ MZB cells were FACS-sorted from spleens of *Cd19*^*Cre/*+^ and *Zbtb24*^*B−CKO*^ mice (male, 14 weeks old), labeled with CFSE (10 μM), and then cultured (~ 1 × 10^5^ cells/well in 96-U bottom plate) with LPS (0.1/0.5 μg/ml), αIgM (5 μg/ml) in the absence/presence of exogenous Hemin (25 μM). On day 3, cells were collected, stained with anti-CD138 before flow cytometry analysis. **A**, representative overlayed histograms showing the cell division profiles (CFSE) of cultured control versus *Zbtb24*^*B−CKO*^ FOB (top) and MZB (bottom) cells. Numbers indicate the percentages of divided CFSE^low^ cells. **B** Bar graphs showing the percentages of divided CFSE^low^ cells in cultured FOB and MZB cells. **C** Representative contour-plots showing the proliferation (CFSE) versus differentiation (CD138) of splenic control (top) versus *Zbtb24*^*B−CKO*^ (bottom) MZB cells in different cultures. **D** Bar graphs showing the percentages CD138^+^ PC cells in differently stimulated MZB cells purified from control versus *Zbtb24*^*B−CKO*^ mice. Each symbol represents a single mouse of the indicated genotype, and numbers below horizonal lines in **B** indicate *P* values determined by student *t*-test. Data are representative of two experiments

Akin to B1 cells, MZB cells are specialized in responses to TID-Ags and are more sensitive, compared to conventional FOB cells, to TLR agonists-induced proliferation (Fig. 8A) and PC differentiation [29]. We thus questioned whether *Zbtb24* exerts a similar function in this innate-like B cell subset as well. LPS stimulation induced robust cell division and PC differentiation of MZB cells, while BCR-crosslinking elicited massive proliferation accompanied by mild PC generation (Fig. 8A–C). Notably, neither the proliferation nor the PC differentiation of LPS-cultivated MZB cells were affected by *Zbtb24* depletion, and the addition of exogenous hemin enhanced PC differentiation of *Zbtb24* deficient or sufficient MZB cells to the same extent (Fig. 8A–D). Hence, depletion of *Zbtb24* does not impact the PC differentiation of MZB cells.

Notably, in stark contrast to the massive PC differentiation but little proliferation of peritoneal B1 cells (Fig. 5E), the vigorously LPS-elicited differentiation of MZB cells was accompanied by extensive cell division (Fig. 8A–C). Moreover, depletion of *Zbtb24* resulted in a subtle but nevertheless consistent decrease of divided CFSE^low^ MZB cells in anti-IgM-stimulated cultures (Fig. 8A, B). This slightly reduced BCR-elicited proliferations of MZB cells may contribute to the decreased antibody levels in *Zbtb24*^*B−CKO*^ mice after TID-Ag immunization as well. We speculated that the distinct metabolic adaptation and energy/substrate allocation between proliferation and differentiation in B1 versus MZB cells, triggered by different stimuli, might underlie the distinct effects of *Zbtb24* in these cells.

## Discussion

The clinical and immunological presentations of *ZBTB24*-disrupted patients with ICF2 imply a prominent role of ZBTB24 in the terminal differentiation and antibody secretion of human B lymphocytes in vivo [3, 44], which prompted us to investigate the cell autonomous role of ZBTB24 in B cell development, differentiation and function by using the conditional knockout mice. Unexpectedly, our data demonstrate that B cell *Zbtb24* is virtually dispensable for TD-Ag-induced GC reactions and antibody responses in vivo, but its deficiency specifically impedes the PC differentiation B1 cells and thereby reduces TID-Ag-induced antibody productions. Although it is thought that the recurrent infections observed in *ZBTB24*-deficient patients with ICF2 mainly stem from the lack of memory humoral responses, our study indicates that defected B1 cell function may contribute to their commonly occurred respiratory and gastrointestinal infections. Thus, ZBTB24 may represent an important regulator in B1 cell-mediated barrier defenses and immune regulations. In addition, our data suggest that ZBTB24 regulate humoral responses in vivo, especially those directed against TD-Ags, beyond the hematopoietic system in mice.

Despite the reduced levels of serum antibodies and peripheral CD19^+^CD27^+^ Bm cells, total amounts of circulating T and B lymphocytes are normal in most ICF cases [3, 44]. Moreover, detailed anatomical and immunological examinations revealed the absence of GC structure in one patients with ICF2 devoid of ZBTB24, and downregulating its expression inhibits the proliferation of a human B cell line by indirectly repressing PRDM1 and IRF4 in vitro [3, 19, 20]. Given the essential role of GC in the propagation of Bm and antibody-secreting PCs [26], these findings strongly indicate that ZBTB24 controls in vivo antibody responses through regulating the GC structure/reaction rather than the general development/maintenance of lymphocytes in humans. In partial agreement of this notion, specific deletion of *Zbtb24* in B cell compartment from the CD19^+^ pro-B stage onward (*Cd19-Cre*) has no effect on the later development, maintenance and phenotype of B cells in the BM and periphery of mice. However, contrary to the Bm and antibody defects in patients with *ZBTB24*-disrupted ICF2, neither the GCB numbers, nor the primary/secondary antibody responses elicited by TD-Ags are significantly decreased in *Zbtb24*^*B−CKO*^ mice, regardless of antigen doses and adjuvant types used for immunization. In keeping with these *in-vivo* findings, *Zbtb24*-depletion does not impair the in vitro survival, activation, proliferation and differentiation of FOB cells, the main responders to TD-Ags.

Apart from B cells, many other immune cells, such as CD4^+^ Th cells and antigen-presenting cells (macrophages and dendritic cells etc.), participate in TD-Ag-induced antibody responses as well. Thus, it is possible that *Zbtb24* modulates humoral responses against TD-Ags in a B cell extrinsic manner. However, mice with a specific depletion of *Zbtb24* in T cells (*Cd4-Cre*) or the whole hematopoietic lineages (*Vav1-Cre*) exhibit intact TD-Ag-induced antibody responses in vivo as well, indicating that hematopoietic *Zbtb24* is dispensable for B cell development/maintenance and the canonical TD-Ag-induced antibody responses via GC reactions in mice. The latter is clearly distinct from patients with *ZBTB24*-disrupted ICF2 with hypo- or a-gammaglobulinemia and lack of circulating Bm cells [3, 44]. The discrepancies in (memory) humoral responses between patients with ICF2 and aforementioned *Zbtb24*-deficient mice may stem from the distinct functions of ZBTB24 across species as evidenced by the fact that *Zbtb24*-null embryo is only viable in humans but not in mice [8]. It is also possible that ZBTB24 may regulate TD-Ag-induced GC reaction and antibody responses beyond the hematopoietic system, for instance, through supporting the formation of proper B cell follicular networks that steers the GC output [50]. In addition, considering the prominent intellectual defects of patients with ICF2 and the recently discovered neuron-spleen axis in regulating GC responses [3, 51], germline *Zbtb24*-deficiency might impair humoral responses against TD-Ags via disrupting the neuron-immune crosstalk as well. Nonetheless, a functional substitution of ZBTB24 by other ZBTB proteins, albeit unlikely based on our RNA-Seq data in splenic B cells, could not be completely excluded.

By contrast, our data highlight an important role of B cell *Zbtb24* in humoral responses against TID-Ags in vivo*.* Upon immunization with a type I or type II TID-Ag, *Zbtb24*^*B−CKO*^ mice generate significantly less antibody-secreting PCs in the presence of normal amounts of B1, FOB, and MZB cells. At the cellular level, depletion of *Zbtb24* markedly restrains the ability of B1 cells to differentiate into PCs and produce antibodies both in vitro upon LPS stimulation and in vivo after transfer into *Rag2*^*−/−*^ recipients without impairing their in vitro survival, activation and proliferation. MZB cells are the other main responders, beyond B1 cells, to TID-Ags, and these two innate-like B cell subsets behave similarly in many aspects. For example, both B1 and MZB are hypersensitive to TLR agonists-induced PC differentiation [29]. Strikingly, *Zbtb24*-ablation does not markedly impact the proliferation and PC differentiation of MZB cells. Therefore, our study demonstrates that B1 cells are the main target B cell subset through which ZBTB24 augments TID-Ag-elicited antibody productions mainly by promoting PC differentiation in vivo. Of note, despite the comparable B1 numbers in naive *Zbtb24*^*B−CKO*^ mice and the little impact of *Zbtb24*-depletion on the survival, activation and anti-CD40-triggered proliferation of B1 cells in vitro, the reduced numbers of *Zbtb24*-null B1 cells in spleens of *Rag2*^*−/−*^ recipients implies that the latter’s long-term survival/maintenance is impaired in some circumstances and thereby contributes to reduced antibody levels in vivo.

Unlike conventional FOB cells that need to undergo extensive proliferation before terminal differentiation, B1 cells do not respond readily to BCR-induced clonal expansion, possibly owing to the highly expressed inhibitory co-receptors like CD5 and LYN. Instead, B1 cells secrete natural IgM at steady state in the absence of antigenic stimulation, and upon encountering with non-specific inflammatory/pathogen-associated stimuli, they promptly and vigorously differentiate into antibody (mainly IgM)-secreting cells [29, 48, 52]. These polyreactive IgM not only participate in the maintenance of tissue homeostasis and prevention of autoimmune reactions, but also constitutes an important first-line defense against infections before activation of the adaptive immune system by facilitating the clearance of autologous dead/dying cells or invading pathogens, respectively [53]. Moreover, B1 cells may regulate the immune response independent of antibody secretion. Both at steady state and after activation, B1 cells may function as a sort of regulatory B (Breg) cells by producing copious amounts of anti-inflammatory cytokine IL-10 [54]. *Zbtb24*-deficient B1 cells produce less IgM and IL-10, it is thus tempting to speculate that the dysfunctional B1 cells contribute to the commonly-occurred infections as well as the uncommonly-observed autoimmune manifestations in patients with ICF2 [44], albeit that the putative surface markers defining human B1 cells remain controversial so far [55]. As such, B cell intrinsic roles of *Zbtb24* in these aspects, such as infections of *S*. *pneumoniae* where B1 cells are involved [56], warrant further studies.

The promptly upregulated PC differentiation and antibody-producing ability of B1 cells upon activation imply that they are transcriptionally and metabolically well prepared for the high secretory capacity. Indeed, innate-like B1 and MZB cells constitutively express more PRDM1 while less BCL6, and are bioenergetically more active than conventional FOB cells [29, 32]. Moreover, sustained mTORC1 signaling coordinates an immediate UPR-affiliated transcriptome profile preceding the expression of XBP1, and confers MZB cells the ability to undergo rapid and robust PC differentiation even in the presence of cell cycle inhibitors [57, 58], which mirrors the unique mitosis-independent PC differentiation of B1 cells as we showed here. In activated conventional splenic B cells, mTORC1 promotes heme synthesis partially by reducing intracellular ROS, and thereby augments their PC differentiation because heme induces PRDM1 by inactivating its repressor molecule BACH2 [35, 37, 38]. Once synthesized, heme enhances mTORC1 activity, possibly through PRDM1 induction or modulating the mitochondrial function [35, 59]. We here showed that mTORC1 exerts a similar function in B1 cells as rapamycin-mediated mTORC1 inhibition greatly inhibits the PC differentiation and heme accumulation in B1 cells as well. Given that *Zbtb24*-depletion reduces mTORC1 activity and PPIX content, and that hemin supplementation almost completely reverts the differentiation defects of *Zbtb24*-null B1 cells even in the presence of rapamycin, our data reveal that ZBTB24 promotes the PC differentiation of B1 cells mainly via heme synthesis, which is partially mediated through mTORC1. Detailed mechanisms underlying the regulations of mTORC1 activity and heme synthesis in B1 cells by ZBTB24 merit further investigation.

Both human and murine Bm cells possess an enhanced heme signature compared to naive B cells, and supplementation of hemin promotes their differentiation toward PCs [59]. Thus, increased heme biosynthesis seems to be a conserved feature of B cells with accelerated and augmented PC differentiation potential, including B1, MZB, and Bm cells. The comparable antibody levels between control and *Zbtb24*^*B−CKO*^ mice after secondary TD-Ag challenge indicate that ZBTB24 has little effect on heme metabolism and PC differentiation of murine Bm cells. Why ZBTB24 only influences the heme biosynthesis and PC differentiation of B1, but not other B cells, at least in mice, is intriguing. Although MZB and B1 cells are considered innate-like B cells and able to mount rapid antibody responses against invading pathogens before the adaptive immune responses are properly propagated and matured, these two cell types are functionally distinct at many aspects. For instance, our data demonstrate that only B1 cells are able to voluntarily uncouple PC differentiation from cell division upon activation, albeit that MZB cells may differentiate into PCs in the presence of cell cycle blockers [58]. It is thus conceivable that B1 cells, depending on the types of stimulation (i.e., LPS versus anti-CD40 triggering), adopt distinct metabolic pathways to devote most of the anergy and biomolecule building blocks either to the PC differentiation/antibody secretion pathway or to the mitosis process. We hypothesize that in case of the PC differentiation pathway, B1 cells immensely and sharply elevate heme biosynthesis, and ZBTB24 somehow participates in this metabolic adaptation process partially by regulating mTORC1 activity in an AKT-independent fashion. In MZB cells, this metabolic reprogramming is much milder and thus less affected by *Zbtb24* deficiency. Of note, despite that mice deficient in *Hmox1*, the heme degrading enzyme, have increased serum antibody levels, administration of hemin to mice does not augment antibody responses against TD- or TID-Ags [38, 59, 60], indicating a complex effect of heme in vivo.

During the preparation of this manuscript, Chen’s group reported that ablation of *Zbtb24* in B cell compartment (*Cd19-Cre*) had no effect on basal serum antibody levels and TD-Ag-induced GC reactions in mice [61], corroborating our findings in this aspect. They found that *Zbtb24*-depletion upregulates *Il5ra* expression, which results in augmented CD19 activity and impaired MZB proliferation induced by BCR [61]. In partial agreement with their findings, BCR-triggered proliferation of MZB cells were impaired upon *Zbtb24*-depletion in our hands, albeit that the defect was far milder. Moreover, our RNA-Seq and/or WB analyses reveal no upregulations in *Il5ra* expression and phosphorylation of CD19 in our *Zbtb24*-depleted B cells (Figs. S11C and S13 and Table S2). These discrepancies may relate to different strategies used to generate the conditional knockout mice. In addition, given the similar phenotypes (largely intact/significantly impaired antibody responses against TD/TID-Ags, respectively) between our *Cd19*^*Cre/*+^ versus *Zbtb24*^*B−CKO*^ mice (both contain one *Cd19* allele) or control versus *Vav1*^*Cre/*+^*Zbtb24*^*loxp/loxp*^ mice (both contain two *Cd19* alleles), it is unlikely that heterogeneous lack of *Cd19* compensates for the loss of *Zbtb24* and thereby results in the grossly normal functions of *Zbtb24*-depleted B2 cells in our study. Nonetheless, we here extend the role of *Zbtb24* in humoral immune response by showing that lack of ZBTB24 significantly impedes the mTORC1 activity, heme synthesis and PC differentiation of peritoneal B1 cells, leading to diminished TID-Ag-induced antibody productions in vivo. Thus, ZBTB24 may represent an important factor involved in regulating barrier defenses against pathogens through B1 cells, and impaired function of B1 cells likely contributes to the clinical presentations of patients with ICF2.

## Conclusions

Our study reveals that ZBTB24 represents a key molecule that specifically promotes the plasma cell differentiation of B1 cells via heme synthesis, at least in mice, which is relevant for barrier defenses against invading pathogens in our bodies. Our study also suggests that defected B1 functions contribute to recurrent infections in patients with ICF2.

## Supplementary Information

**Additional file 1: Table S1.** gRNA and primer sequences used in this study; **Figure S1.** Little impact of *Zbtb24*-deficiency on the phenotype of B cells in the periphery of mice; **Figure S2.** No effect of *Zbtb24*-deficiency on the phenotype and number of GCB cells after TD-Ag immunization; **Figure S3.** Deficiency of *Zbtb24* in B cells has no impact on the long-term antibody responses in mice after NP_19_-OVA/alum immunization; **Figure S4.** Reduced total antibody-producing ability of *Zbtb24*^*B−CKO*^ splenic B cells after adoptive transfer; **Figure S5.** Grossly intact CSR abiltiy of *Zbtb24*^*B−CKO*^ B cells; **Figure S6.** Little effect of *Zbtb24*-deficiency in hematopoietic cells on humoral responses against TD-Ags in mice. **Figure S7.** Comparable numbers of NP^+^ B cells in *Cd19*^*Cre/*+^ and *Zbtb24*^*B−CKO*^ mice on D35 after NP-Ficoll immunization; **Figure S8.** Reduced numbers of NP^+^ B1 cells in *Zbtb24*^*B−CKO*^ mice after NP-LPS immunization; **Figure S9.** *Zbtb24*-deficiency has no impact on the differentiation and antibody-producing ability of splenic B cells in vitro; **Figure S10.** *Zbtb24*-deficiency does not compromise the survival, activation and proliferation of *Zbtb24*^*B−CKO*^ B cells in vitro; **Figure S11.** Dysregulated genes/signaling pathways in *Zbtb24*-deficient B1 cells; **Figure S12.** No effect of *Zbtb24*-deficiency on heme metabolism in stimulated splenic B cells. **Figure S13.** Little impact of *Zbtb24*-depletion on the phosphorylation of CD19, AKT and ribosomal protein S6 (S6) in splenic B cells.

**Additional file 2: Table S2.** Selected gene expressions in RNA-Seq analysis of splenic B cells.

**Additional file 3:** Uncropped WB membranes.

## Data Availability

The RNA-Seq data were deposited in the GEO database with the accession numbers GSE241746 and GSE241747. The majority of data supporting the findings of this study are presented within the article and its Supplemental Material. Data that are not directly included are available from the lead corresponding author (Dr. Jun Wang) upon reasonable request.

## Abbreviations

ICF: Immunodeficiency, centromeric instability, and facial anomalies syndrome
GC: Germinal center
GCB: GC B cells
Tfh: T follicular helper cells
Bm: Memory B cells
PCs: Plasma cells
CSR: Class-switch recombination
BM: Bone marrow
FOB: Follicular B cells
MZB: Marginal zone B cells
TD-Ags: T cell dependent antigens
SHM: Somatic hypermutation
BCR: B cell receptor
TD-Ags: T cell independent antigens
TFs: Transcriptional factors
*Zbtb24*^*B*^^*−*^^*CKO*^: B cell specific *Zbtb24* knockout mice (*Cd19*^*Cre/*^^+^*Zbtb24*^*loxp/loxp*^)
WT: Wild type
PPIX: Protoporphyrin IX
