# Supplementary material for: The ICF2 gene Zbtb24 specifically regulates the differentiation of B1 cells via promoting heme synthesis
Source: Cell Mol Biol Lett. 2024 Sep 14;29:123. doi: 10.1186/s11658-024-00641-2 (PMC11401330; doi:10.1186/s11658-024-00641-2)
Supplement: Supplementary file 1 — Additional file 1: Table S1. gRNA and primer sequences used in this study; Figure S1. Little impact of Zbtb24-deficiency on the phenotype of B cells in the periphery of mice; Figure S2. No effect of Zbtb24-deficiency on the phenotype and number of GCB cells after TD-Ag immunization; Figure S3. Deficiency of Zbtb24 in B cells has no impact on the long-term antibody responses in mice after NP19-OVA/alum immunization; Figure S4. Reduced total antibody-producing ability of Zbtb24B−CKO splenic B cells after adoptive transfer; Figure S5. Grossly intact CSR abiltiy of Zbtb24B−CKO B cells; Figure S6. Little effect of Zbtb24-deficiency in hematopoietic cells on humoral responses against TD-Ags in mice. Figure S7. Comparable numbers of NP+ B cells in Cd19Cre/+ and Zbtb24B−CKO mice on D35 after NP-Ficoll immunization; Figure S8. Reduced numbers of NP+ B1 cells in Zbtb24B−CKO mice after NP-LPS immunization; Figure S9. Zbtb24-deficiency has no impact on the differentiation and antibody-producing ability of splenic B cells in vitro; Figure S10. Zbtb24-deficiency does not compromise the survival, activation and proliferation of Zbtb24B−CKO B cells in vitro; Figure S11. Dysregulated genes/signaling pathways in Zbtb24-deficient B1 cells; Figure S12. No effect of Zbtb24-deficiency on heme metabolism in stimulated splenic B cells. Figure S13. Little impact of Zbtb24-depletion on the phosphorylation of CD19, AKT and ribosomal protein S6 (S6) in splenic B cells. [file 11658_2024_641_MOESM1_ESM.pdf]

Table S1. gRNA and primer sequences used in this study.

Name	Sequence (5'→3')	Application
***Zbtb24*-gRNA1**	CCGGAGGGCCGTACAGACTTTTG	loxp1 insertion
***Zbtb24*-gRNA2**	CCAGCCTCACGTAGACACTTTAT	loxp2 insertion
***Zbtb24*-screen F2**	TAACGGACCGGAGGGCGTCG	ES clone selection
***Zbtb24*-screen R3**	TCAAGTGATAAAGTGTCTACGTGAGGTGCTA	ES clone selection
***Zbtb24*-WT Fw**	CCATCTTGTAAATATCTTCTGGGTGTCT (in WT allele)	Genotyping (*Zbtb24*)
***Zbtb24*-WT Rv**	GATAAAGTGTCTACGTGAGGCTGGG (in WT allele)	Genotyping (*Zbtb24*)
***Zbtb24*-CKO Rv**	GTGTCTACGTGAGGTGCTATAACTTCG (in loxp allele)	Genotyping (*Zbtb24*)
***Cd19-Cre*-M Fw**	GCGGTCTGGCAGTAAAAACTATC (in Cre allele)	Genotyping (*Cd19-Cre*)
***Cd19-Cre*-M Rv**	GTGAAACAGCATTGCTGTCACTT (in Cre allele)	Genotyping (*Cd19-Cre*)
***Cd19-Cre*-W Fw**	CCTCTCCCTGTCTCCTTCCT (in WT allele)	Genotyping (*Cd19-Cre*)
***Cd19-Cre*-W Rv**	TGGTCTGAGACATTGACAATCA (in WT allele)	Genotyping (*Cd19-Cre*)
**mZBTB24 Fw**	CCTTGTGGGCAGCTTATGGT	RT-qPCR (ZBTB24)
**mZBTB24 Rv**	CAAGGCTTTATGGGCTCGGA	RT-qPCR (ZBTB24)
**mCPOX Fw**	GAGGAAGCTGACGGTAACACA	RT-qPCR (CPOX)
**mCPOX Rv**	CCCGCGGTGAACTATAAAGA	RT-qPCR (CPOX)
**mALAD Fw**	GCCTCCTGAGTGAAAATGGA	RT-qPCR (ALAD)
**mALAD Rv**	CAGAGACCCTGTTGCCAAGT	RT-qPCR (ALAD)
**mGAPDH Fw**	AACTTTGGCATTGTGGAAGG	RT-qPCR (GAPDH)
**mGAPDH Rv**	GGATGCAGGGATGATGTTCT	RT-qPCR (GAPDH)

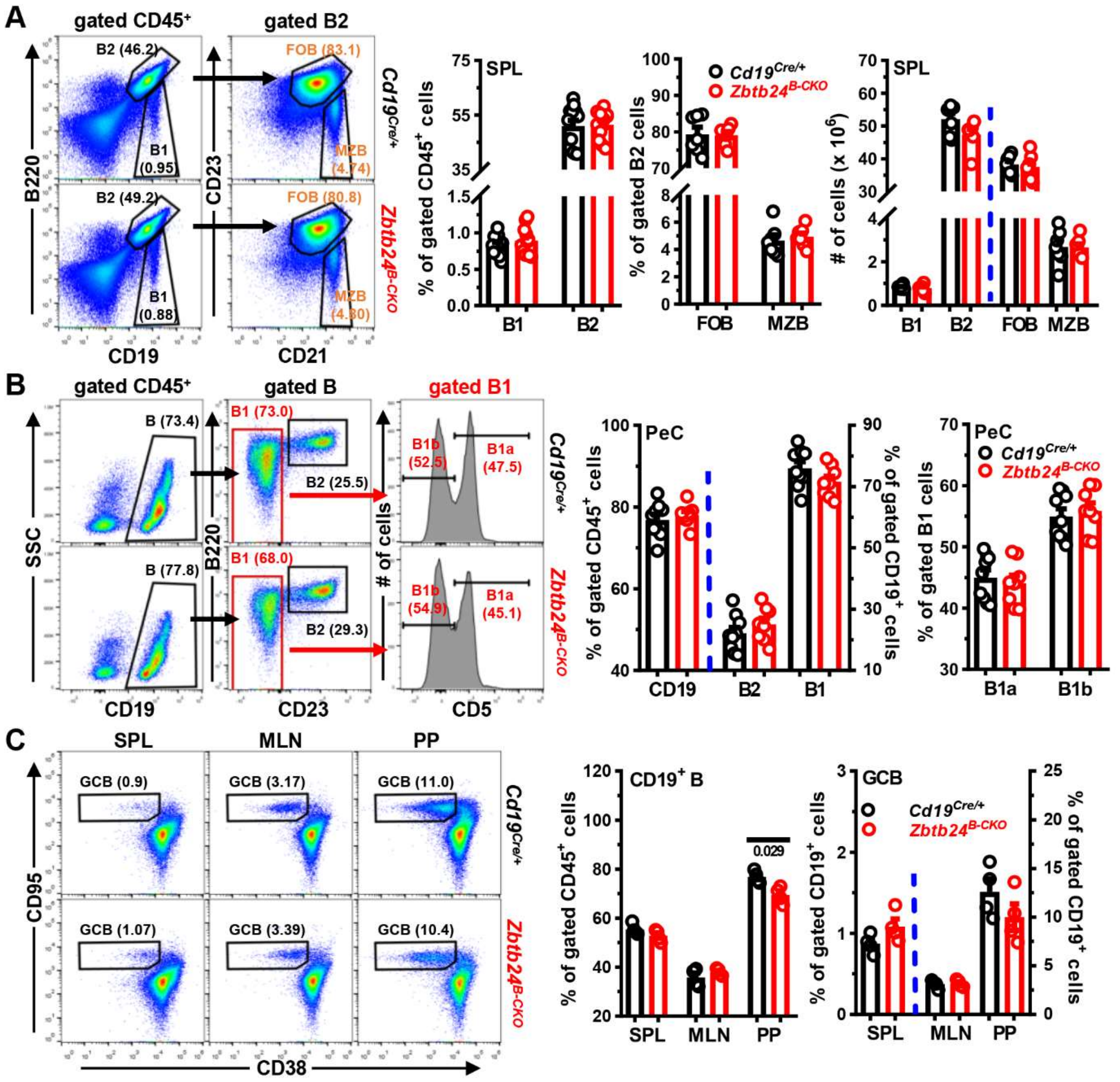

Figure S1. Little impact of *Zbtb24*-deficiency on the phenotype of B cells in the periphery of mice. Cells from the spleens (SPL), peritoneal cavities (PeC), mesenteric lymph nodes (MLN) and Peyer's patches (PP) of mice (female, 8-10 weeks old) were stained with antibodies against the indicated surface molecules before flow cytometry analyses. **A&B**, representative pictures/bar graphs showing the gating strategies/percentages of B2 ($CD19^{+}B220^{high}$ in SPL or $CD19^{+}B220^{high}CD23^{+}$ in PeC, respectively), B1 ($CD19^{+}B220^{low}$ in SPL or $CD19^{+}B220^{low}CD23^{-}$ in PeC, respectively), B1a/b ($CD5^{+}$/$CD5^{-}$, respectively, within gated B1), follicular (FOB)/marginzal zone (MZB) ($CD23^{high}CD21^{low}$/$CD23^{low}CD21^{high}$, repectively, within gated B2) in the SPL (**A**) or PeC (**B**) of *Cd19*$^{Cre/+}$ and *Zbtb24*$^{B\text{-}CKO}$ mice. The absolute cell numbers are also shown in the far-right panel of **A**. **C**, representative pseudo-plots/bar graphs showing the gating strategies/percentages of germinal center B (GCB, $CD95^{high}CD38^{low}$) within gated $CD19^{+}$ B cells in the SPL, MLN and PP of *Cd19*$^{Cre/+}$ & *Zbtb24*$^{B\text{-}CKO}$ mice. Each symbol represents a single mouse of the indicated genotype. The absolute numbers of indicated B-cell subsets did not differ significantly in the PeC, MLN and PP of the two groups of mice (data not shown). AU, arbitrary units. Pooled data from two-independent experiments were shown in **A&B**.

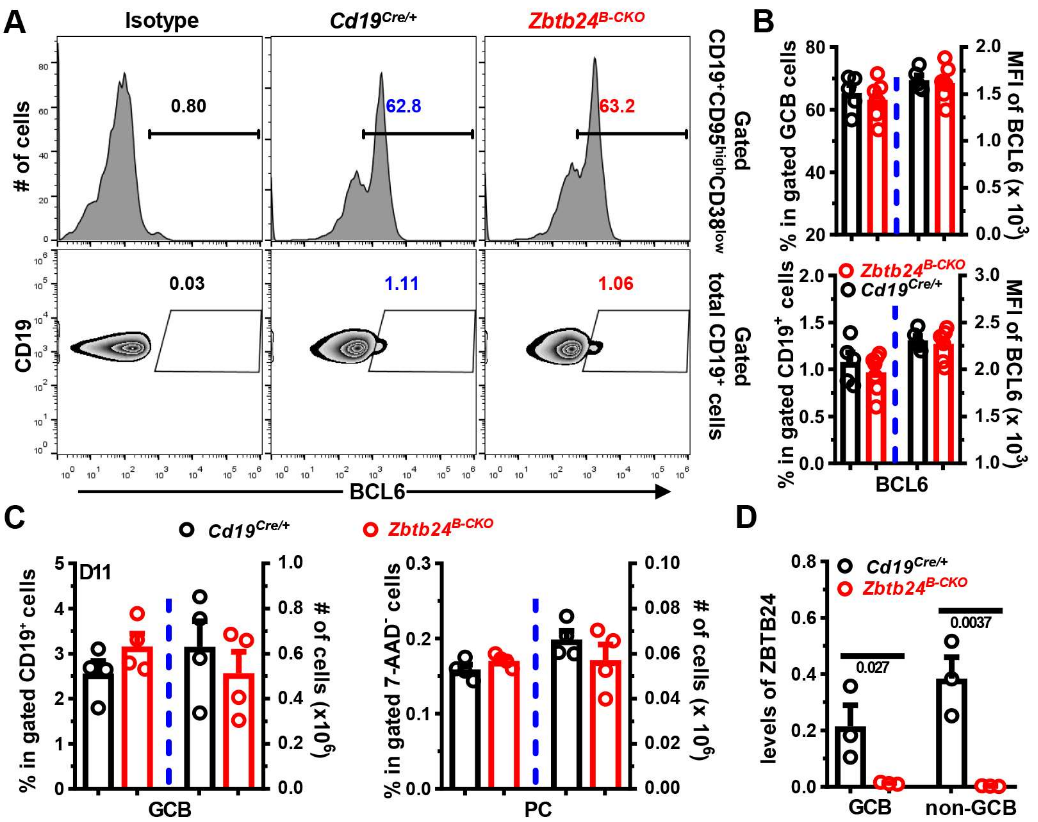

Figure S2. No effect of *Zbtb24*-deficiency on the phenotype and number of GCB cells after TD-Ag immunization. Splenic cells in mice were stained with antibodies against CD19/CD95/CD38 & BCL6 (intracellular) or CD19/CD138 plus 7-AAD to visualize the percents/phenotypes of GCB or PC cells in mice depicted in Figure 2A-C on D14 post NP_{19}-OVA/IFA immunization (**A&B**) or on D11 post i.p. immunization with sheep red blood cells (SRBC, $1x10^9$ cells/200 µl PBS/mouse, female, 8 weeks of age) (**C&D**). **A**, representative histograms (*upper panel*) or zebra-plots (*lower panel*) showing the percentages of $BCL6^+$ cells in gated $CD19^+CD95^{high}CD38^{low}$ GCB cells (*upper panel*) or total $CD19^+$ B cells (*lower panel*) in control *Cd19*$^{Cre/+}$ and Zbtb24$^{B\text{-}CKO}$ mice. **B**, bar graphs showing the percentages of $BCL6^+$ cells/MFI of BCL6 in gated $CD19^+CD95^{high}CD38^{low}$ GCB cells (*upper panel*) or total $CD19^+$ B cells (*lower panel*). **C**, bar graphs showing the percentages/absolute numbers of $CD19^+CD95^{high}CD38^{low}$ GCB and $CD19^{low}CD138^{high}$ PC cells in spleens of mice on D11 post immunization. **D**, mRNA levels of ZBTB24 in FACS-purified $CD19^+CD95^{high}CD38^{low}$ GCB and $CD19^+CD95^-CD38^+$ non-GCB cells from spleens of immunized mice. Each symbol represents a single mouse of the indicated genotype, and numbers below horizon lines indicate *P* values determined by student *t*-test.

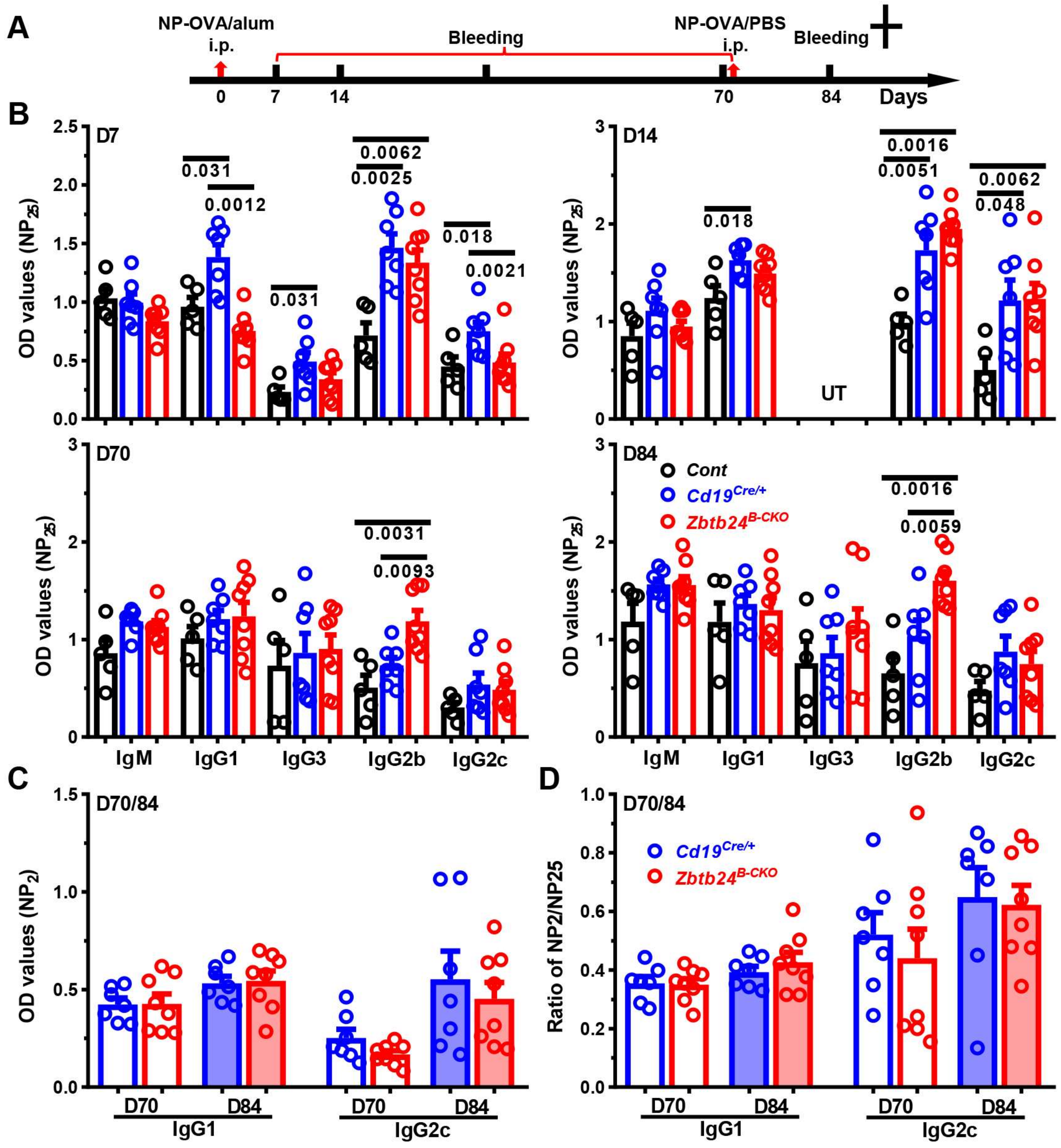

Figure S3. Deficiency of *Zbtb24* in B cells has no impact on the long-term antibody responses in mice after NP$_{19}$-OVA/alum immunization. Mice (female, 8 weeks of age) were i.p. immunized with alum-precipitated NP$_{19}$-OVA (NP-OVA/alum, 100 μg/100 μl/mouse) on D0, and rechallenged with NP$_{19}$-OVA in PBS (50 μg/100 μl/mouse) on D70. **A**, a schematic diagram depicting the experimental setup. **B&C**, bar graphs showing the optical density (OD) values of NP-specific antibody subtypes against coated NP$_{25}$-BSA (NP$_{25}$, **B**) or NP$_2$-BSA (NP$_2$, **C**) in diluted sera of *Zbtb24*$^{loxp/loxp}$ (*Cont*), *Cd19*$^{Cre/+}$ and *Zbtb24*$^{B\text{-}CKO}$ mice on indicated days. **D**, bar graphs showing the ratios of relatively high-affinity (NP$_2$) to low-affinity (NP$_{25}$) NP-specific IgG1 & IgG2c in *Cd19*$^{Cre/+}$ and *Zbtb24*$^{B\text{-}CKO}$ mice on D70 & D84. Each dot represents a single mouse of the indicated genotype and numbers below horizonal lines indicate *P* values determined by Mann-Whitney test. UT, untested.

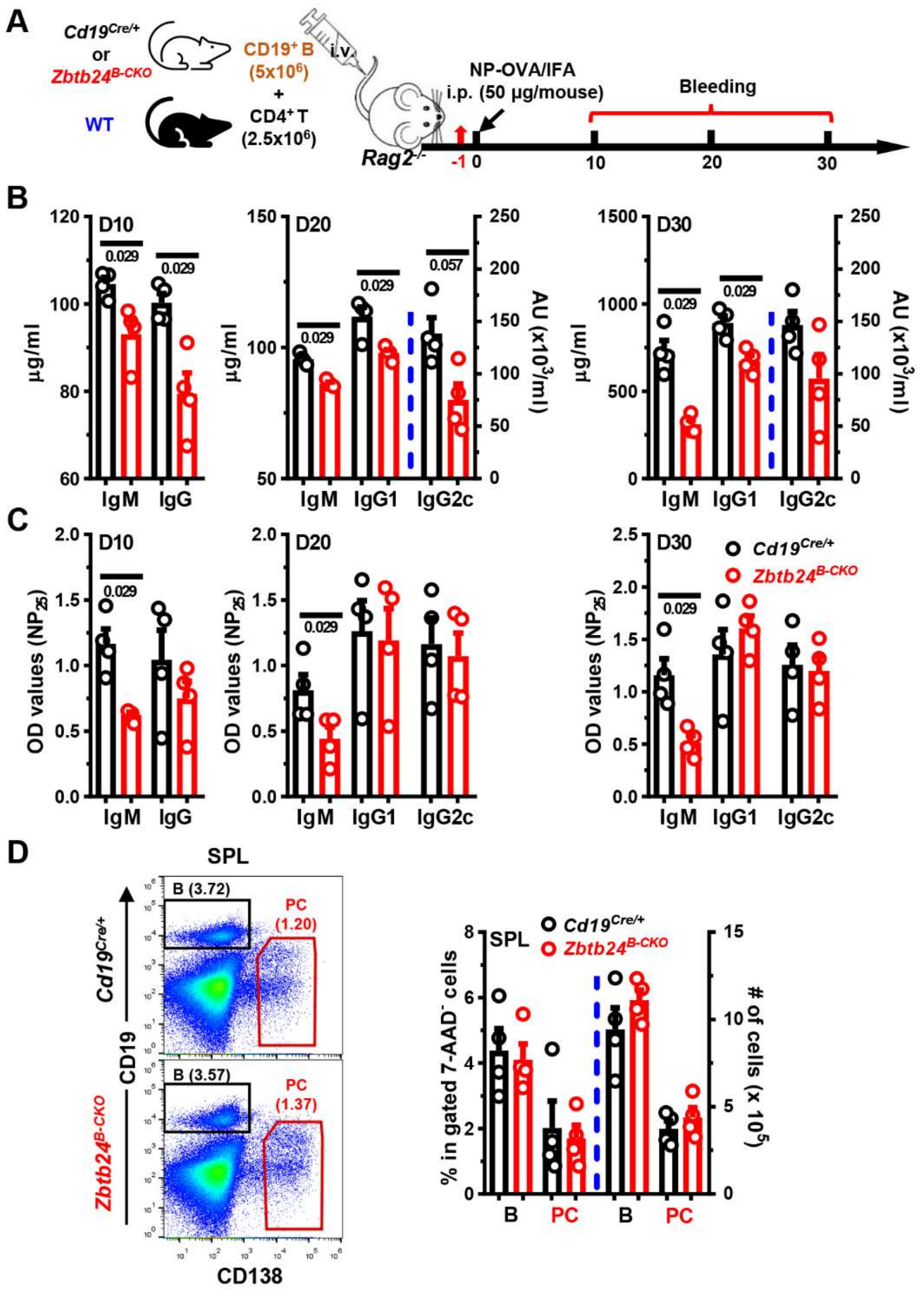

Figure S4. Reduced total antibody-producing ability of *Zbtb24$^{B\text{-}CKO}$* splenic B cells after adoptive transfer. Total CD19^{+} B cells, enriched from *Zbtb24$^{B\text{-}CKO}$* or control *Cd19$^{Cre/+}$* mice (male, 8 weeks of age) by magnetic beads, were mixed with CD4^{+} T cells (purified from male WT mice) at the ratio of 2:1 before being intravenously (i.v.) injected into the *Rag2$^{-/-}$* recipient mice (5x10^{6} B cells plus 2.5x10^{6} T cells per mouse). One day later, recipient mice were immunized with NP$_{19}$-OVA emulsified in IFA (NP-OVA/IFA) intraperitoneally (i.p.). Blood was taken at indicated times, and total or NP-specific antibody levels in sera were determined by ELISA. **A**, a schematic flow-chart of the experiment setup. **B&C**, bar graphs showing levels of total (**B)** or NP-specific (**C)** IgM/IgG or IgG1/IgG2c levels in sera of recipient mice at indicated times. **E**, bar graphs showing the percentages and absolute numbers of CD19^{+} B cells or CD19$^{-/low}$CD138^{+} plasma cells (PC) in the spleens (SPL) and bone marrows (BM) of *Cd19$^{Cre/+}$ vs. Zbtb24$^{B\text{-}CKO}$* mice. Representative pseudo-plots showing the gates for each population were shown in **D**. Each dot represents a single recipient mouse, and numbers below horizontal lines indicate *P* values. AU, arbitrary units.

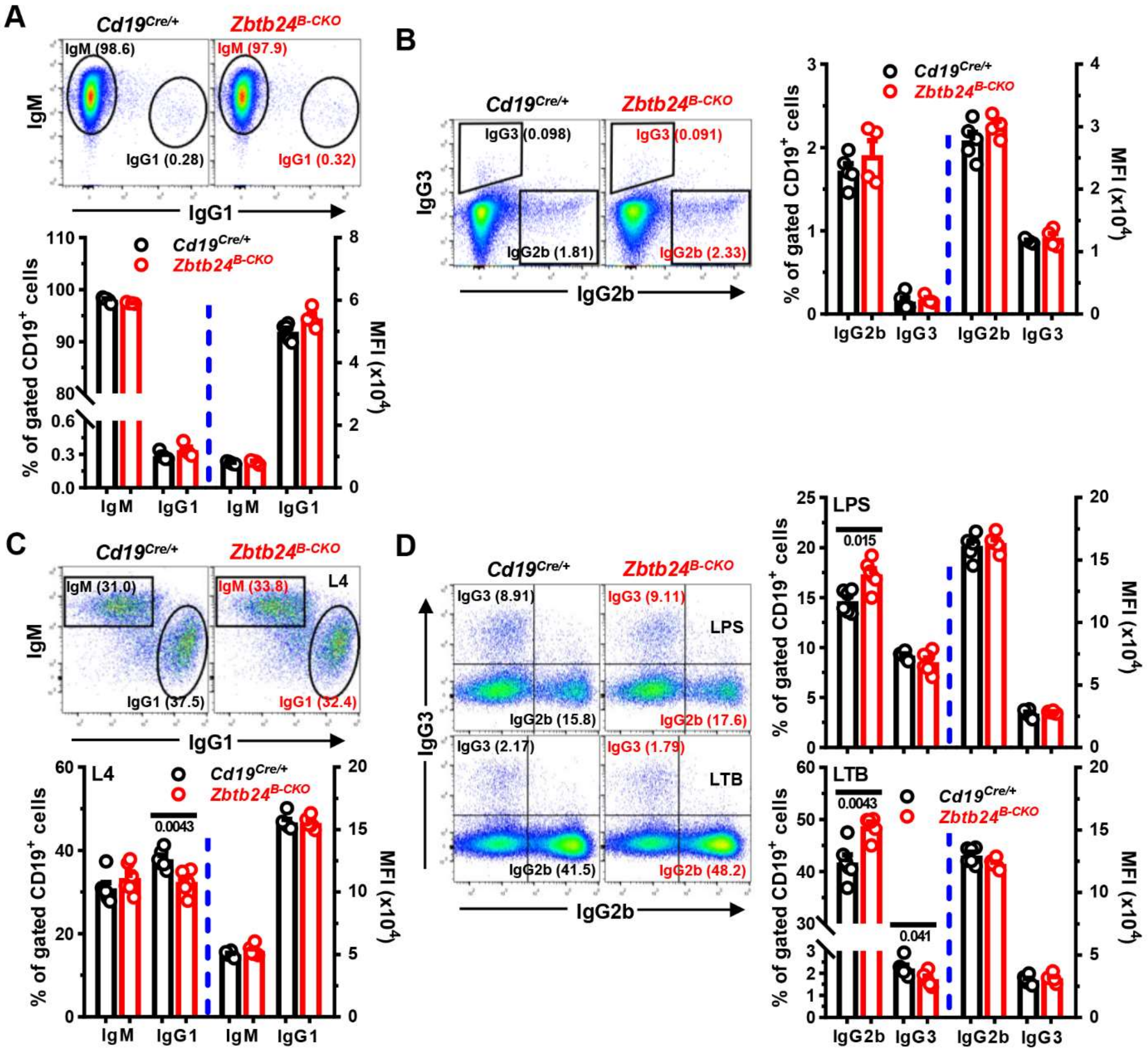

Figure S5. Grossly intact CSR abiltiy of *Zbtb24*$^{B\text{-}CKO}$ B cells. Splenic B cells (2 x 10^5 cells/well) from *Cd19*$^{Cre/+}$ or *Zbtb24*$^{B\text{-}CKO}$ mice were stimulated with LPS (10 μg/ml) in the absence/presence of IL-4 (25 ng/ml) or TGF-β (1 ng/ml) plus BAFF (10 ng/ml) in 96 U-bottom plates. On day 4, cells were collected and expressions of surface IgM/IgG2b/IgG3 & IgG1 were analyzed by flow cytometry. **A&C,** representative pseudo-plots and bar graphs showing the surface IgM/IgG1 on B cells before (**A**) and after culture with LPS plus IL-4 (L4, **C**). **B&D**, representative pseudo-plots/bar graphs showing the surface IgG2b/IgG3 on B cells before (**B**) and after culture with LPS without (*upper panel* in **D**)/with TGF-β & BAFF (LTB, *lower panel* in **D**). Each symbol represents a single mouse of the indicated genotype (female, 10 weeks of age), and numbers below horizontal lines indicate *P* values. MFI, mean fluorescence intensity.

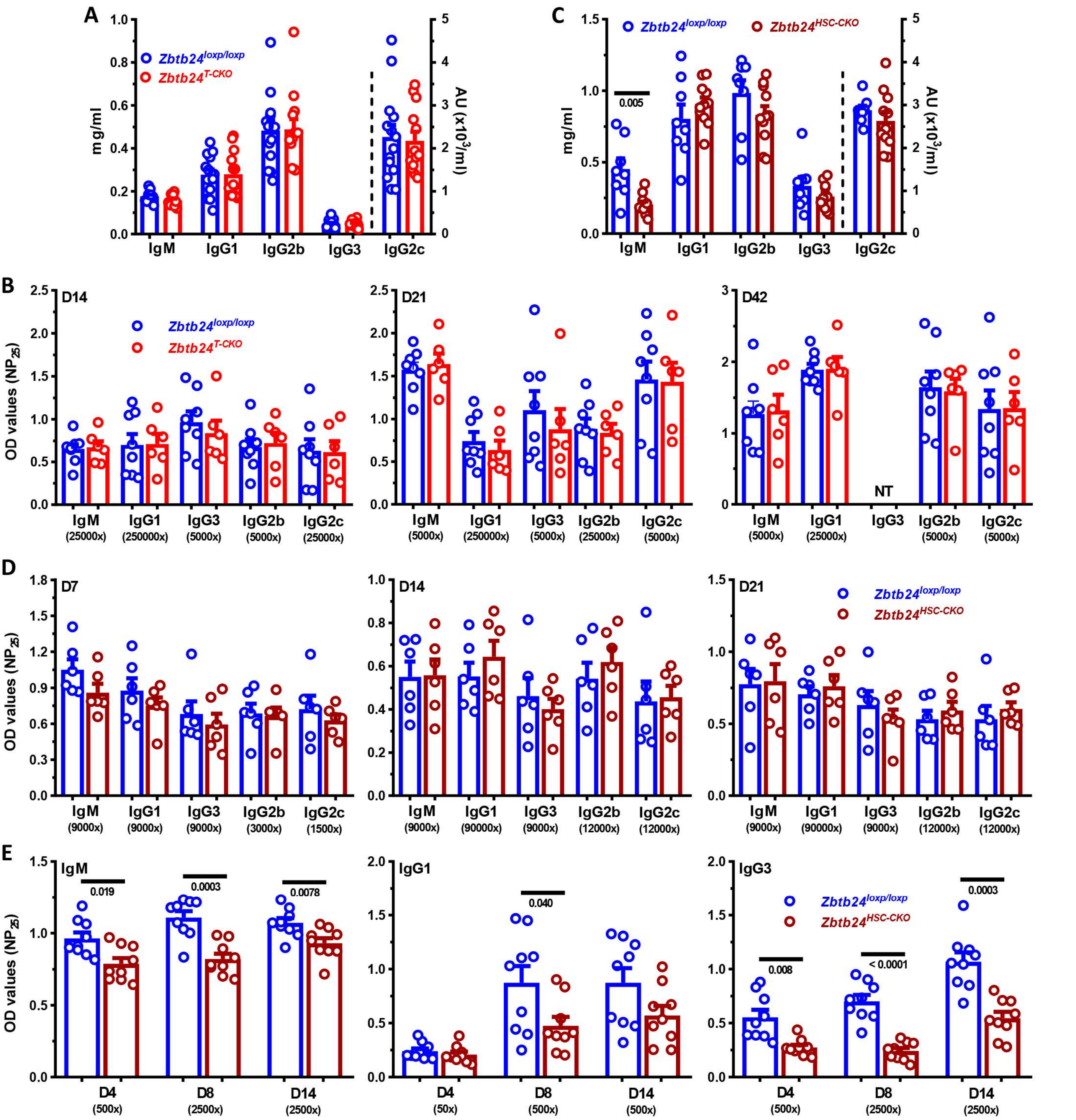

Figure S6. Little effect of *Zbtb24*-deficiency in hematopoietic cells on humoral responses against TD-Ags in mice. A&C, bar graphs showing levels of total IgM, IgG1, IgG2b, IgG2c and IgG3 in sera of naive $Zbtb24^{loxp/loxp}$ *vs.* $Zbtb24^{T\text{-}CKO}$ ($Cd4^{Cre/+}Zbtb24^{loxp/loxp}$) mice (**A**, female, 10-12 weeks old) or naive $Zbtb24^{loxp/loxp}$ *vs.* $Zbtb24^{HSC\text{-}CKO}$ ($Vav1^{Cre/+}Zbtb24^{loxp/loxp}$) mice (**C**, male, 8-10 weeks old). Deletion of *Zbtb24* in the hematopoietic system does not significantly impact the phenotypes and numbers of T and B cells in the peripheral lymph organs of mice (data not shown). **B, D&E**, bar graphs showing the optical density (OD) values of NP-specific antibody subtypes against coated NP_{25}-BSA (NP_{25}) in diluted sera of $Zbtb24^{loxp/loxp}$ *vs.* $Zbtb24^{T\text{-}CKO}$ (**B**, male, 8-10 weeks old) or $Zbtb24^{loxp/loxp}$ *vs.* $Zbtb24^{HSC\text{-}CKO}$ mice (**D&E**) on indicated days after i.p. immunizations with NP_{19}-OVA emulsified in IFA (**B**, 1:1, 30 µg/100 µl mouse), alum-precipitated NP_{25}-CGG (**D**, 25 µg/100 µl/mouse, 8-10 weeks old males) or NP-Ficoll (**E**, 10 µg/100 µl/mouse, 8-week old males) on D0. Each dot represents a single mouse of the indicated genotype. Data were pooled from two independent experiments. Numbers below horizonal lines indicate *P* values determined by Mann-Whitney test, and numbers in brackets below the X-axis denote the dilution factor for each antibody subtype. NT, not tested.

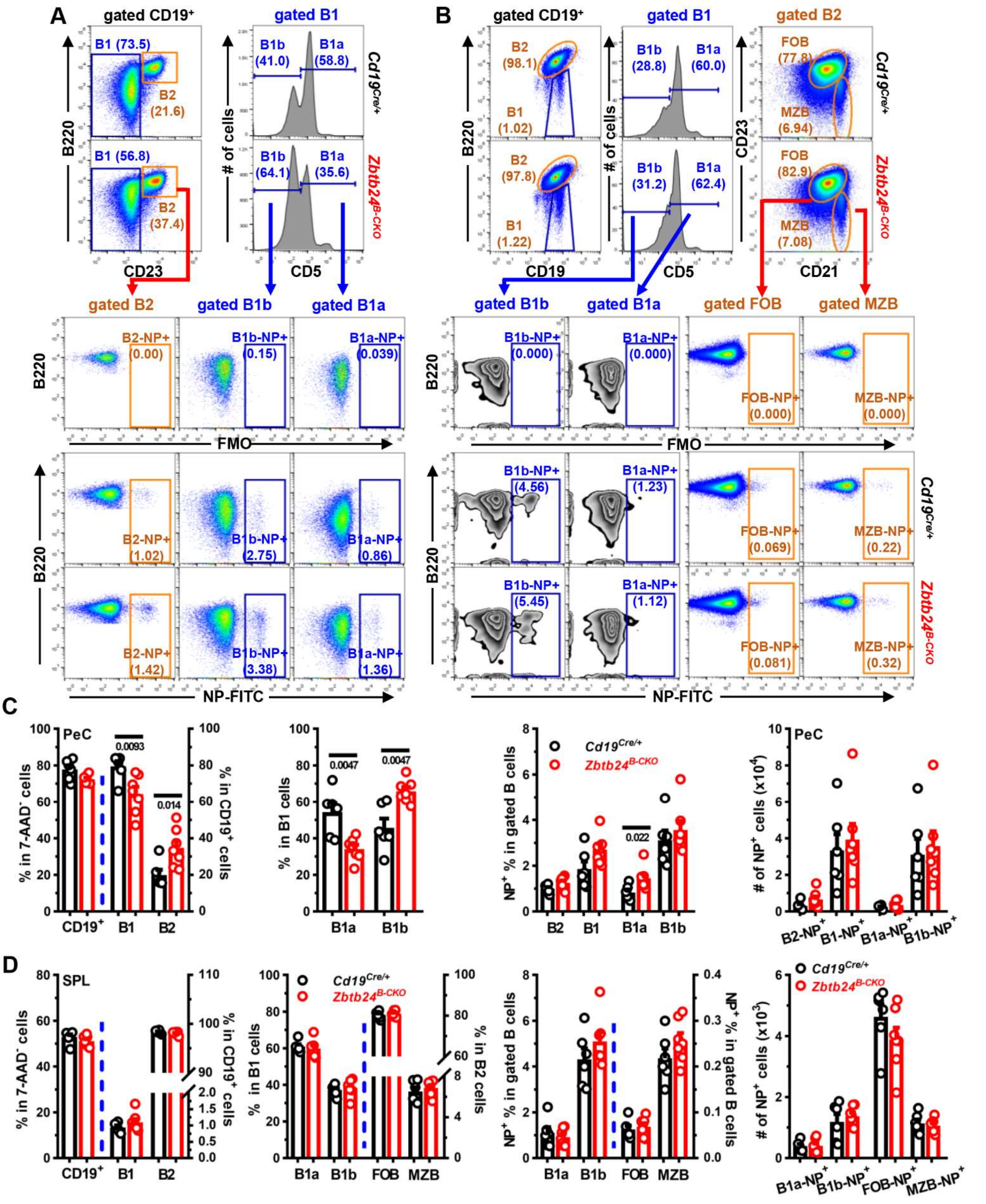

Figure S7. Comparable numbers of NP$^+$ B cells in *Cd19$^{Cre/+}$* & *Zbtb24$^{B\text{-}CKO}$* mice on D35 after NP-Ficoll immunization. Peritoneal and splenic cells in NP-Ficoll immunized mice (depicted in Figure 3A-E) were stained with antibodies against B220/CD19/CD5/CD21/CD23 in combination with 7-AAD and NP-FITC. **A&B**, representative flow cytometry plots showing the gating strategies to identify NP$^+$ cells within gated B2/B1, B1a/B1b & follicular (FOB)/marginal zone (MZB) B cells in peritoneal cavities (**A**) and spleens (**B**). Gates for NP$^+$ cells within B2 (B2-NP+), B1b (B1b-NP+), B1a (B1a-NP+), FOB (FOB-NP+) & MZB (MZB-NP+) were set based on the FMO (fluorescence minus one/FITC-channel) samples. **C&D**, bar graphs showing the percentages/absolute numbers of NP$^+$ cells within indicated B-cell subsets in peritoneal cavities (PeC, **C**) and spleens (SPL, **D**) of *Cd19$^{Cre/+}$* & *Zbtb24$^{B\text{-}CKO}$* mice. Each dot represents a single mouse of the indicated genotype, and numbers below horizonal lines indicate *P* values.

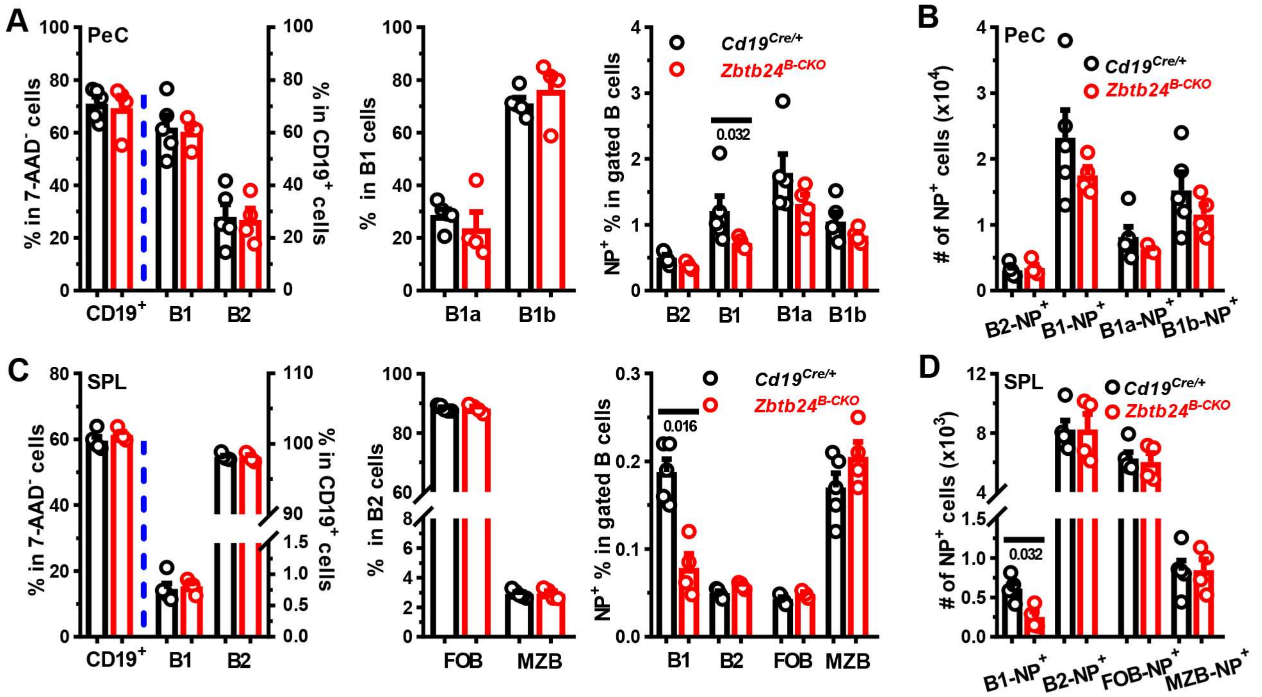

Figure S8. Reduced numbers of NP^{+} B1 cells in *Zbtb24$^{B\text{-}CKO}$* mice after NP-LPS immunization. Peritoneal and splenic cells in NP-LPS immunized mice (depicted in Figure 3F-H) were stained and analyzed as described in Figure S7A&B. **A&B**, bar graphs showing the percentages (**A**) and absolute numbers (**B**) of NP^{+} cells within indicated B-cell subsets in peritoneal cavities (PeC) of mice. **C&D**, bar graphs showing the percentages (**C**) and absolute numbers (**D**) of NP^{+} cells within indicated B-cell compartments in spleens (SPL) of *Cd19$^{Cre/+}$ vs. Zbtb24$^{B\text{-}CKO}$* mice. Each dot represents a single mouse of the indicated genotype, and numbers below horizonal lines indicate *P* values.

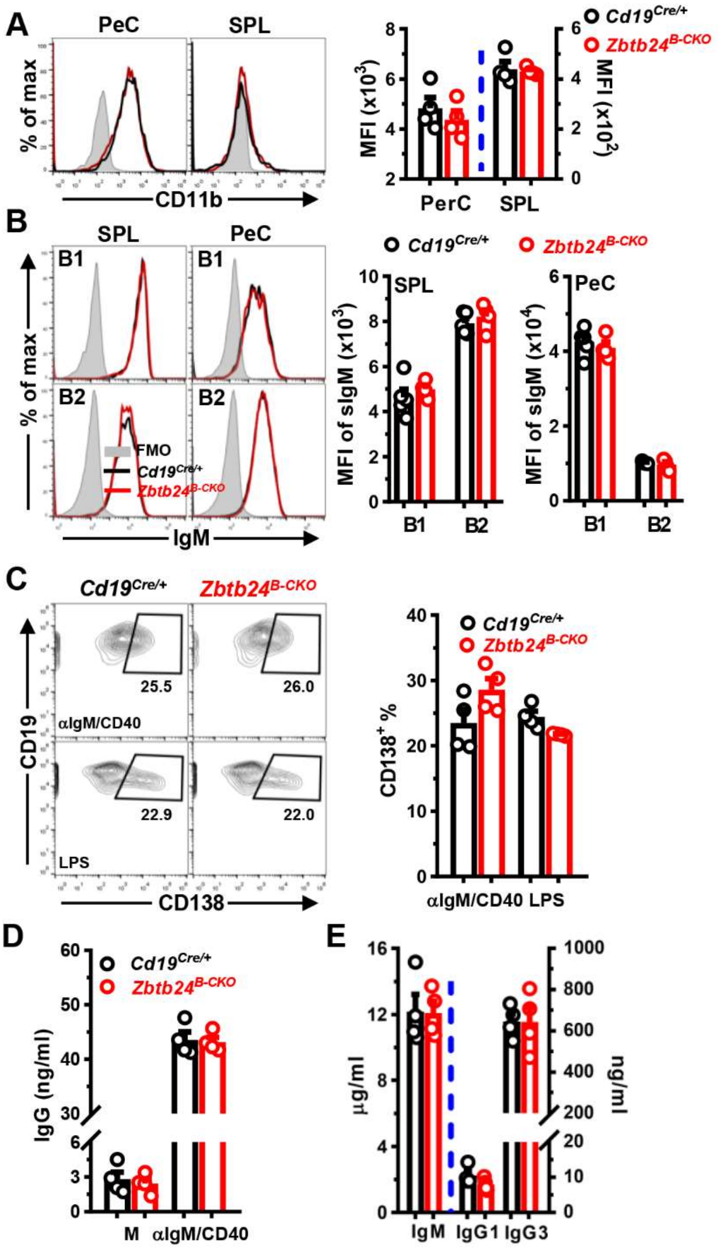

Figure S9. *Zbtb24*-deficiency has no impact on the differentiation and antibody-producing ability of splenic B cells *in vitro*. A&B, representative overlayed-histograms & bar graphs showing levels of surface CD11b (**A**) or IgM (sIgM, **B**) on splenic (SPL)/peritoneal (PeC) B1/B2 cells in *Cd19*$^{Cre/+}$ *vs. Zbtb24*$^{B\text{-}CKO}$ mice. Splenic and peritoneal cells were stained with antibodies against CD19/B220/CD23 & CD11b/IgM before flow cytometry analyses. **C**, representative contour-plots & bar graphs showing percentages of CD138^{+} PC cells in cultured splenic CD19^{+} B cells. **D&E**, levels of IgG (**D**) or IgM/IgG1/IgG3 (**E**) in supernatants of splenic B cells cultured in M, αIgM/CD40 (**D**) or LPS (**E**). CD19^{+} B cells were FACS-sorted from spleens of *Cd19*$^{Cre/+}$ & *Zbtb24*$^{B\text{-}CKO}$ mice, and subsequently cultured (1 x 10^5 cells/well in 96-well plate) in medium (M), anti-IgM & anti-CD40 (αIgM/CD40, 5/5 μg/ml) or LPS (10 μg/ml). On day 4, surface expressions of CD138 on cultured B cells were analyzed by flow cytometry, and antibody levels in culture supernatants were determined by ELISA (**C-E**). Each symbol represents a single mouse of the indicated genotype (female, 8-10 weeks of age).

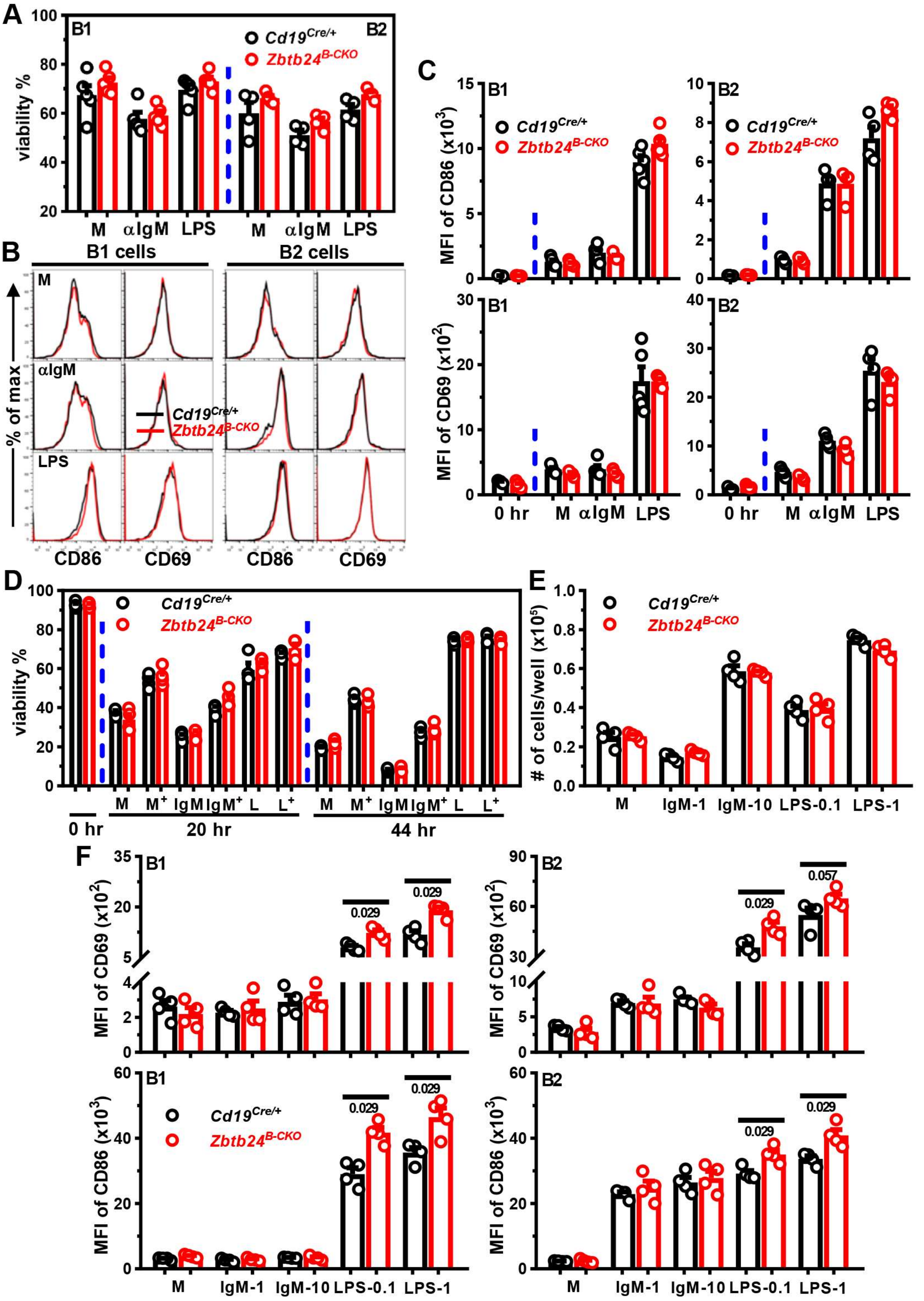

Figure S10. *Zbtb24*-deficiency does not compromise the survival, activation and proliferation of *Zbtb24$^{B\text{-}CKO}$* B cells *in vitro*. CD19$^+$B220^{low}CD23$^-$ B1 and CD19$^+$B220^{high}CD23$^+$ B2 cells were FACS-sorted from peritoneal cavities of *Cd19$^{Cre/+}$* & *Zbtb24$^{B\text{-}CKO}$* mice, and subsequently cultured (2-5 x 10^4 cells/well in 96-U bottom plate) in medium (M), αIgM (2 μg/ml) or LPS (0.5 μg/ml). Viabilities (based on forward/side scatter) and surface levels of CD69/CD86 were analyzed by flow cytometry (**A-C**). **A**, bar graphs showing viabilities of peritoneal B1 & B2 cells at 24 hrs. **B&C**, representative overlayed histograms (**B**) or bar graphs (**C**) showing surface expressions of CD86 & CD69 on 24-hr cultured peritoneal B1/B2 cells derived from *Cd19$^{Cre/+}$ vs. Zbtb24$^{B\text{-}CKO}$* mice before (0 hr) and after 24 hr's culture. CD19$^+$ B cells were purified from spleens of *Cd19$^{Cre/+}$* & *Zbtb24$^{B\text{-}CKO}$* mice, and subsequently cultured (1.5-2 x 10^5 cells/well in 96-well plate) in medium, anti-IgM or LPS before flow cytometry analysis (**D-F**). **D**, bar graphs showing the percents of living cells (7-AAD$^-$ & Annexin V$^-$) before (0 hr) or after cultures in M, 1 μg/ml anti-IgM (IgM) or LPS (L) in the absence/presence of 0.2 μg/ml BAFF (indicated by a '+' in the upper-right corner) for 20 & 44 hrs. **E**, bar graphs showing the numbers of living cells (based on forward/side scatter) in different cultures on day 3. **F**, bar graphs showing the expression levels of surface CD69 (top row) and CD86 (bottom row) on gated B2 (CD19$^+$B220^{high}, left column) or B1 (CD19$^+$B220^{low}, right column) cells after 20 hrs' culture in medium (M), 1/10

μg/ml anti-IgM (IgM-1/IgM-10, respectively), 0.1/1 μg/ml LPS (LPS-0.1/LPS-1, respectively). Each symbol represents a single mouse of the indicated genotype (12-week-old males in **A-C**, and 8-week-old females in **D-F**), and numbers below horizontal lines in **F** indicate *P* values.

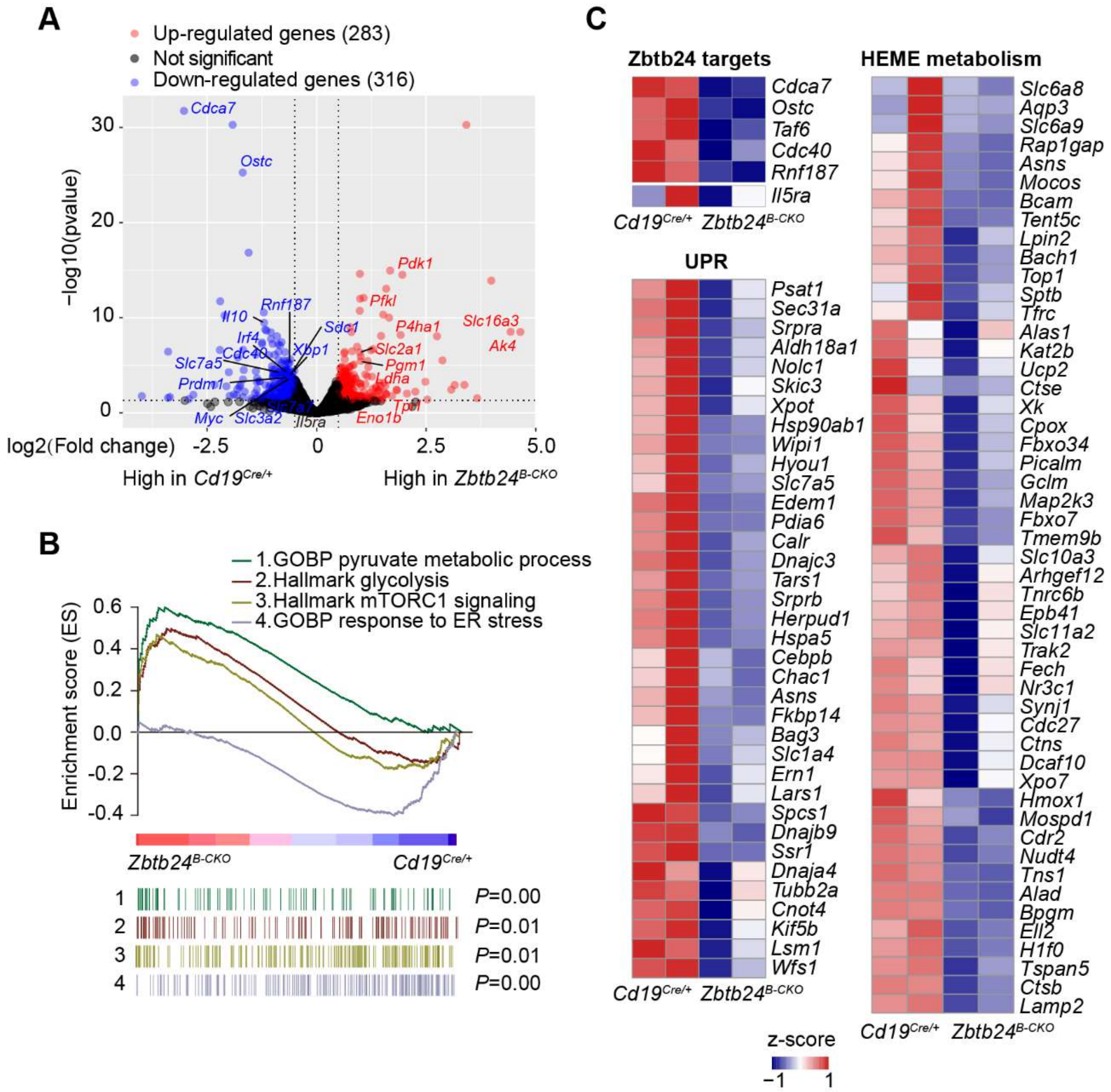

Figure S11. Dysregulated genes/signaling pathways in *Zbtb24*-deficient B1 cells. FACS-sorted peritoneal CD19^{+}B220^{low}CD23^{-} B1 cells were stimulated with 0.1 μg/ml LPS in 96-U bottom plate for 24 hrs (related to Figure 6A-C). **A**, volcano plots showing upregulated (red) or downregulated (blue) genes in *Zbtb24*$^{B\text{-}CKO}$ B1 cells. **B**, GSEA plots showing the enrichment of genes regulating pyruvate metabolism, glycolysis, mTORC1 signaling and ER stress in LPS-stimulated *Zbtb24*$^{B\text{-}CKO}$ *vs. Cd19*$^{Cre/+}$ B1 cells. **C**, heatmaps showing the z-score normalized on the raw expression counts of reported ZBTB24 target genes or those affiliated to UPR and heme metabolism in *Zbtb24*-deficient B1 cells by GSEA.

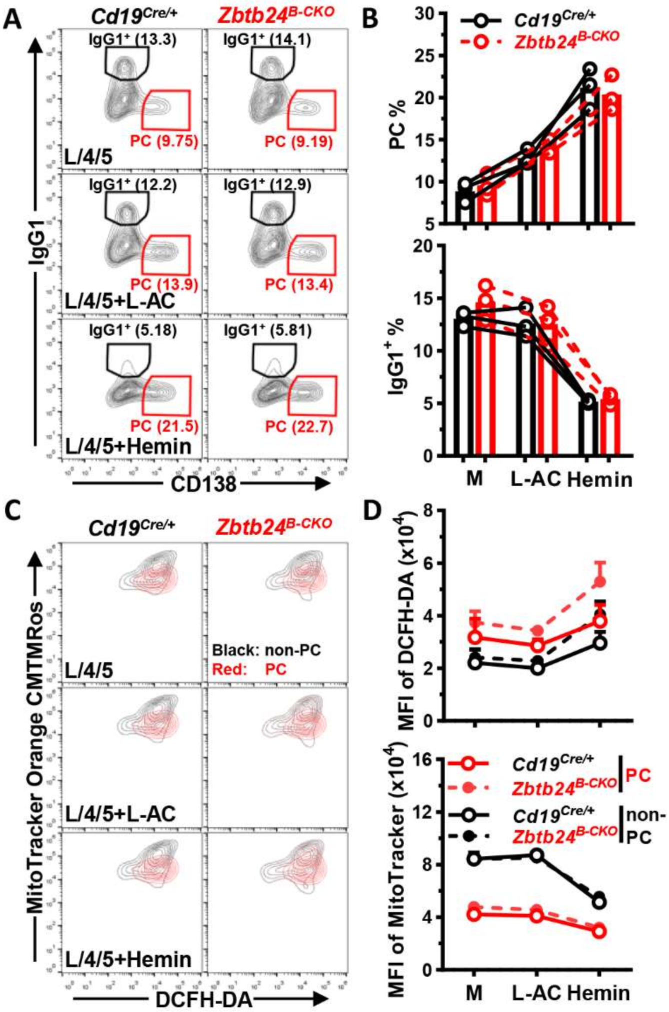

Figure S12. No effect of *Zbtb24*-deficiency on heme metabolism in stimulated splenic B cells. Splenic B cells, isolated from $Cd19^{Cre/+}$ or $Zbtb24^{B\text{-}CKO}$ mice, were stimulated with 10 µg/ml LPS plus 10 ng/ml IL-4 & IL-5 (L/4/5) without/with additional L-AC (250 µM) or Hemin (25 µM) in 96-U bottom plate for 4 days. **A&B**, representative contour-plots (**A**) and bar graphs (**B**) showing the percentages of class-switched $CD138^-IgG1^+$ B cells ($IgG1^+$) or differentiated $IgG1^-CD138^+$ PC in stimulated $Cd19^{Cre/+}$ *vs.* $Zbtb24^{B\text{-}CKO}$ splenic B cells on day 4. **C**, representative overlayed contour-plots showing the intracellular ROS levels (visualized by DCFH-DA) and mitochondrial mass/membrane potentials (detected by MitoTracker Orange CMTMRos) in gated $CD138^-$ non-PC (black) *vs.* $CD138^+$ PC (red) in cultured splenic B cells on day 4. **D**, line graphs showing the ROS levels (MFI of DCFH-DA, *upper panel*) and mitochondrial mass/membrane potential (MFI of MitoTracker, *lower panel*) in gated non-PC/PC cells in $Cd19^{Cre/+}$ *vs.* $Zbtb24^{B\text{-}CKO}$ splenic B cell cultures. Each dot in **B** represents a single mouse of the indicated genotype (male, 14 weeks of age), while results in **D** are expressed as mean ± SEM (n=3).

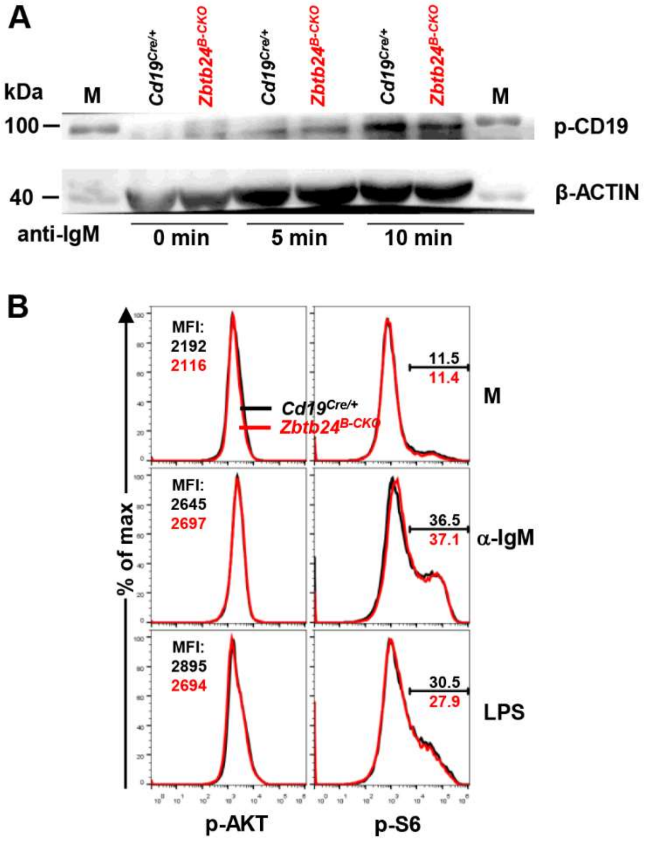

Figure S13. Little impact of *Zbtb24*-depletion on the phosphorylation of CD19, AKT and ribosomal protein S6 (S6) in B cells. Beads-enriched CD19^{+} B cells were activated with anti-IgM (10 μg/ml) for 0/5/10 mins (**A**), or stimulated with medium (M), anti-IgM (10 μg/ml) or LPS (1 μg/ml) for 30 hrs (**B**) *in vitro* before analysis. **A**, levels of phosphorylated CD19 (p-CD19) were detected by WB. M in the far left/right lane denotes marker. **B**, Levels of phosphorylated AKT (p-AKT) and S6 (p-S6, an event controlled by mTORC1 activity) were analyzed by flow cytometry. Experiments were performed twice with similar results.
