# Supplementary material for: The ICF2 gene Zbtb24 specifically regulates the differentiation of B1 cells via promoting heme synthesis
Source: Cell Mol Biol Lett. 2024 Sep 14;29:123. doi: 10.1186/s11658-024-00641-2 (PMC11401330; doi:10.1186/s11658-024-00641-2)
Supplement: Supplementary file 3 — Additional file 3: Uncropped WB membranes. [file 11658_2024_641_MOESM3_ESM.pdf]

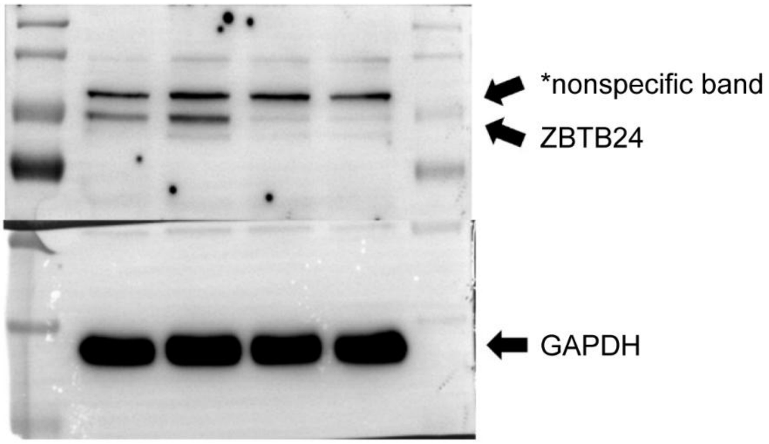

Uncropped picture
of Figure 1D (CD19$^+$ B cells)

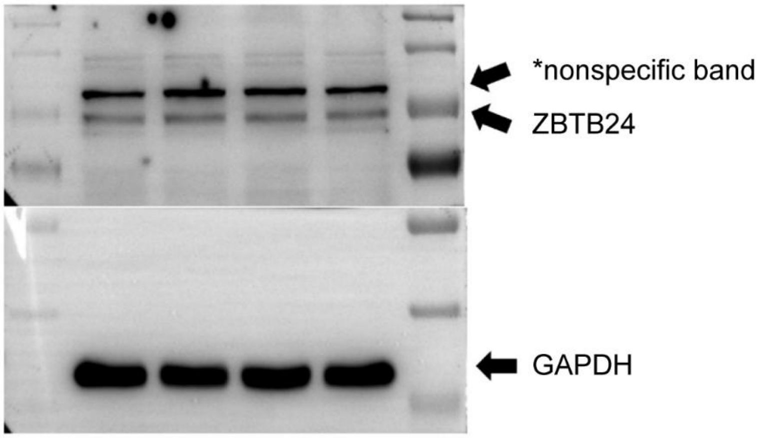

Uncropped picture
of Figure 1D (CD19$^-$ B cells)

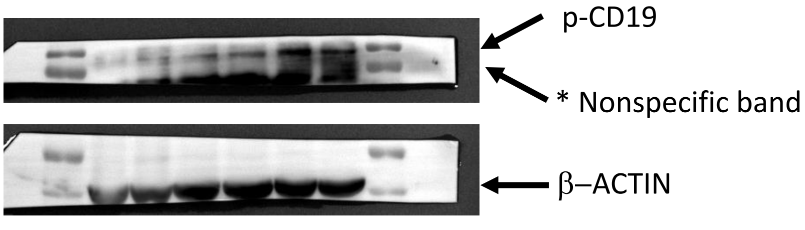

Uncropped picture
Of Figure S13A
